# Mucosal metabolites fuel the growth and virulence of *E. coli* linked to Crohn’s disease

**DOI:** 10.1172/jci.insight.157013

**Published:** 2022-05-23

**Authors:** Shiying Zhang, Xochitl Morgan, Belgin Dogan, Francois-Pierre Martin, Suzy Strickler, Akihiko Oka, Jeremy Herzog, Bo Liu, Scot E. Dowd, Curtis Huttenhower, Matthieu Pichaud, Esra I. Dogan, Jack Satsangi, Randy Longman, Rhonda Yantiss, Lukas A. Mueller, Ellen J. Scherl, R. Balfour Sartor, Kenneth W. Simpson

**Affiliations:** 1 Department of Clinical Sciences, College of Veterinary Medicine, Cornell University, Ithaca, New York, USA.; 2 Department of Microbiology and Immunology, University of Otago, Dunedin, New Zealand.; 3 Nestlé Institute of Health Sciences, Nestlé Research, Société des Produits Nestlé S.A., Lausanne, Switzerland.; 4 Boyce Thompson Institute, Cornell University, Ithaca, New York, USA.; 5 Shimane University Faculty of Medicine, Shimane, Japan.; 6 Department of Medicine, Center for Gastrointestinal Biology and Disease, University of North Carolina (UNC) at Chapel Hill, North Carolina, USA.; 7 Molecular Research LP, Shallowater, Texas, USA.; 8 Biostatistics Department, Harvard T.H. Chan School of Public Health, Boston, Massachusetts, USA.; 9 Enterome, Paris, France.; 10 Translational Gastroenterology Unit, Nuffield Department of Medicine, John Radcliffe Hospital Oxford, United Kingdom.; 11 Jill Roberts Center for Inflammatory Bowel Disease, Weill Cornell Medical College, Cornell University, New York, New York, USA.

**Keywords:** Inflammation, Microbiology, Amino acid metabolism, Inflammatory bowel disease

## Abstract

Elucidating how resident enteric bacteria interact with their hosts to promote health or inflammation is of central importance to diarrheal and inflammatory bowel diseases across species. Here, we integrated the microbial and chemical microenvironment of a patient’s ileal mucosa with their clinical phenotype and genotype to identify factors favoring the growth and virulence of adherent and invasive *E*. *coli* (AIEC) linked to Crohn’s disease. We determined that the ileal niche of AIEC was characterized by inflammation, dysbiosis, coculture of *Enterococcus*, and oxidative stress. We discovered that mucosal metabolites supported general growth of ileal *E*. *coli*, with a selective effect of ethanolamine on AIEC that was augmented by cometabolism of ileitis-associated amino acids and glutathione and by symbiosis-associated fucose. This metabolic plasticity was facilitated by the *eut* and *pdu* microcompartments, amino acid metabolism, γ-glutamyl-cycle, and pleiotropic stress responses. We linked metabolism to virulence and found that ethanolamine and glutamine enhanced AIEC motility, infectivity, and proinflammatory responses in vitro. We connected use of ethanolamine to intestinal inflammation and L-fuculose phosphate aldolase (*fucA*) to symbiosis in AIEC monoassociated *IL10^–/–^* mice. Collectively, we established that AIEC were pathoadapted to utilize mucosal metabolites associated with health and inflammation for growth and virulence, enabling the transition from symbiont to pathogen in a susceptible host.

## Graphical abstract

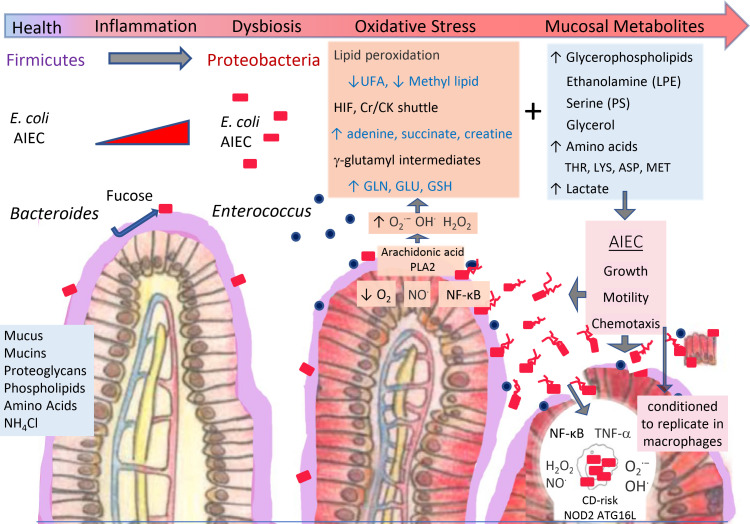

## Introduction

Inflammatory bowel diseases (IBDs) are broadly classified by the region involved and histopathology, either as Crohn’s disease (CD; segmental granulomatous transmural ileitis and/or colitis) or ulcerative colitis (UC; continuous colonic mucosal inflammation). However, these simple classifications fail to account for the complex, genetic, and environmental interactions that manifest clinically as IBD, related comorbidities, and their variable clinical course and response to treatment (1, 2). Mechanistically, IBD is considered a consequence of dysregulated interplay between host genetic susceptibility and immunity, the enteric microenvironment, and environmental triggers, but the specific interactions that lead to IBD are unresolved (3).

The microbial and chemical microenvironment — which varies in the small and large bowel, as well as the lumen and mucosa — has emerged as a key factor influencing bacterial colonization and virulence and influencing the transition from symbiont to pathogen (3–6). The microbiome of ileal CD is characterized by expansion of aggressive Proteobacteria and mucosa-associated *E*. *coli*, relative to depleted protective Firmicutes (7–9). *E*. *coli* that adhere to and invade epithelial cells and persist in macrophages in vitro (adherent and invasive *E*. *coli* [AIEC]), have been isolated from 21%–63% of patients with ileal CD (9–11). AIEC can exploit defects in intracellular killing and autophagy conferred by CD risk polymorphisms in ATG16L, IRGM, and NOD2, and it can elicit Th1/Th17 immune responses in patients with CD (11, 12). Moreover, AIEC is causally associated with granulomatous colitis in dogs (13) and intestinal inflammation in genetically susceptible mice (14, 15).

The factors regulating AIEC abundance in the ileum are ill defined. Paneth cell dysfunction (16), loss of beneficial bacteria (e.g., *Faecalibacterium prausnitzii*; ref. 17), changes in niche, nutrient availability, or other aspects of the environment may be involved (5–7, 9). Studies in murine models of CD have shown, in the absence of genetic susceptibility, that acute ileitis induces the proliferation of AIEC (18), paralleling the ability of enteropathogens to thrive in the inflamed intestine (5, 6). Comparative genomic analyses have identified genes encoding microcompartments (MCPs) used by *Salmonella* and diarrheagenic *E*. *coli* to metabolize fucose; 1,2-propanediol (1,2-PD, *pdu* operon); and ethanolamine (EA, *eut* operon) for growth and energy in the inflamed intestine in 50% of AIEC versus 20% non-AIEC (*pdu*) and 100% of *E*. *coli* (*eut*), respectively (19–22). Functional evaluation of these MCPs in AIEC links the use of 1,2-PD, EA, and downstream metabolites propionate and acetate to growth, colonization, and virulence in preclinical models (19, 21, 23–25). The increased abundance of *E*. *coli* transcripts encoding metabolism of EA (*eutB*, *eutS*) and 1,2-PD (*pduC*) in the luminal contents or feces of patients with CD (21, 25, 26) suggests that inflammation-associated increases in these substrates may fuel the growth and virulence of AIEC in the inflamed intestine (23, 27). However, nonpathogenic *E*. *coli* also utilizes these metabolites, and metabolite use by *E*. *coli* varies in the intestinal lumen and mucosa (27–29). As previous studies did not analyze the ileal mucosal metabolome and resident *E*. *coli* (21, 23, 24), the spectrum of metabolites in the ileal mucosa of patients with and without IBD, ileitis, and AIEC — and the ability of those metabolites to selectively favor the growth and virulence of AIEC — remain to be determined.

Here, we adopt a patient-based approach that combines direct analysis of the microbiome, metabolome, and cultivable bacteria of the ileal mucosa with IBD phenotype and risk-genotype, to guide functional analysis of AIEC and non-AIEC isolated from the same patients. We establish that AIEC is able to utilize mucosal metabolites associated with health and inflammation for growth, energy, stress resistance, and virulence, and we show that metabolic pathoadaptation may underpin the ability of AIEC to transition from symbiont to pathogen in a susceptible host.

## Results

### Patient characteristics, IBD phenotype, and genotype.

To test the hypothesis that clinical, host genetic, microbial and metabolic parameters correlate with culture of ileal AIEC, we established a cohort of 97 patients: 68 with IBD (CD: *n* = 43, 33 ileal, 10 colonic; UC: *n* = 25) and 29 non-IBD (NI: 14 surveillance endoscopy, 15 non-IBD predominantly IBS) (Supplemental Table 1; supplemental material available online with this article; https://doi.org/10.1172/jci.insight.157013DS1). The ileum of patients with CD was significantly more inflamed than that of patients with UC and NI (*P* < 0.05) (Supplemental Table 1). The prevalence of CD risk–associated polymorphisms in NOD2 and ATG16LT300A was similar in CD, UC, and NI (range NOD2, 33%–44% risk; ATG16L, 26%–29%; GG, 48%–59%; A/G, 15%–24% AA; Supplemental Table 1). Patients with ATG16LAG had a higher need for surgery (70%) than ATG16LGG (40%) and AA (29%), and than NOD2- R702W, G908R, 1007FS (53%), but this was not significant in this relatively small cohort (Supplemental Table 2).

### E. coli, Enterococcus, Klebsiella and Streptococcus are the predominant cultivable ileal mucosal bacteria.

**Figure 1:**
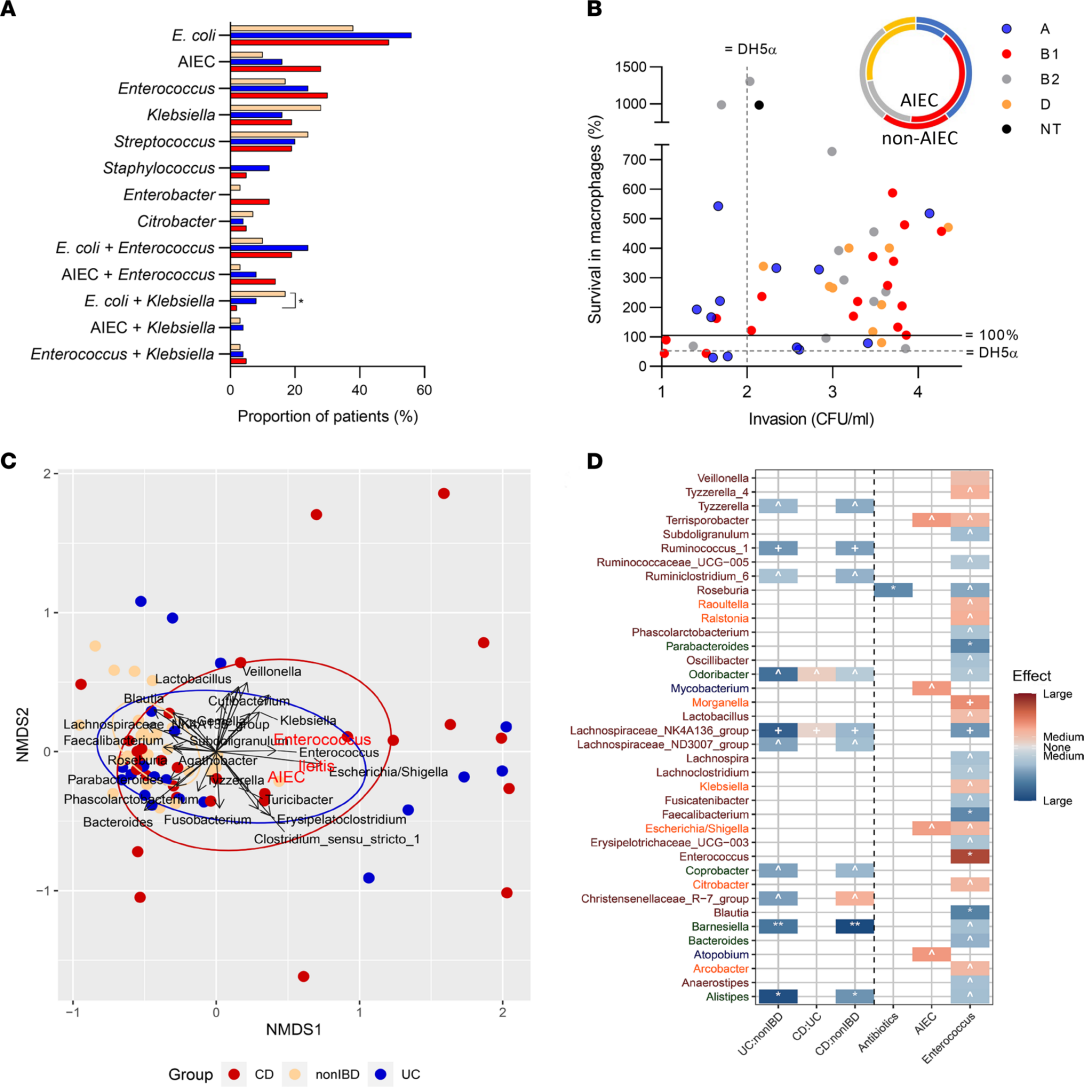
The mucosal microbiome of AIEC shares the dysbiosis and culture of *Enterococcus* observed in a subset of patients with CD and UC. (**A**) Culture of ileal mucosa from patients with CD, UC, and NI. Fisher’s exact test (*P < 0.05, 2-tailed). (**B**) AIEC pathotype in cultured cells by strain and phylogroup (A, B1, B2, D, and nontypeable NT). Spearman correlation, phylogroup B1; *P* = 0.5935 and *P* < 0.05 (2-tailed). (**C**) Nonmetric multidimensional scaling (NMDS) ordination using Bray-Curtis distance. Stress = 0.16. Taxa with a significant fit to the ordination (*P* < 0.05, envfit) are shown. Line length corresponds to strength of association. The centroids of ileitis-positive samples (*P* < 0.001, *R*^2^ = 0.17, PERMANOVA) and AIEC-positive samples (*P* = 0.068, *R*^2^ = 0.05, PERMANOVA) are shown in red. (**D**) Taxa associated with disease phenotype, antibiotic use, and colonization by *Enterococcus* and AIEC. Kruskal-Wallis tests (Benjamini-Hochberg *q* < 0.25) were used to determine taxa with differential abundance between groups of interest. For all significant associations, effect size (η^2^) was calculated. Intensity of color corresponds to magnitude of effect, and color corresponds to direction of effect. Symbols indicate significance of association (**q < 0.01, *q < 0.05, ^+^*q* < 0.1, ^*q* < 0.2). Color on *y* axis labels corresponds to phylum.

Culture of ileal mucosa for aerobic and facultative-anaerobic bacteria yielded *E*. *coli*, *Enterococcus*, *Klebsiella*, *Streptococcus*, *Enterobacter*, *Staphylococcus*, and *Citrobacter* (Figure 1A). There was no significant difference in cultivable species by disease phenotype (*P* > 0.05). The most frequent combinations were *E*. *coli* and *Enterococcus* (UC, 24%; CD 19%, NI 11%; *P* > 0.05), and *E*. *coli* and *Klebsiella* (NI, 18%; UC, 8%; CD, 2%; *P* < 0.05) (Figure 1A). Isolation rates for *Enterococcus* were 1.5–2 times higher (*P* < 0.05) in patients with a history of taking metronidazole compared with those without antibiotics (Supplemental Figure 1A).

### Phylogroup and virulence gene content of mucosal E. coli correlate with IBD phenotype and AIEC pathotype.

We characterized a pool of 615 *E*. *coli* isolates from 52 patients by genotype, phylogroup, and virulence gene content, and we identified 63 strains (median 1, range 1–2/patient) for further study. *E*. *coli* strains from CD patients with ileal (ICD) and colonic (CCD) involvement and NI were distributed across the A, B1, B2, and D phylogroups with similar prevalence of virulence genes (Supplemental Figure 1B and Supplemental Tables 3 and 4). In contrast, *E*. *coli* from UC were enriched in phylogroup B2 (*P* = 0.0047) and ExPEC-associated virulence genes, *malX*, *chuA*, and *colV* (*P* < 0.05; Supplemental Figure 1B and Supplemental Table 3). Thirteen *E*. *coli* strains with gentamicin resistance, diarrheagenic virulence genes, or cytotoxicity were excluded from AIEC pathotyping in cell culture. AIEC were cultured from 28% CD, 17% UC, and 10% NI patients (*P* > 0.05). Patients with a history of taking metronidazole or ciprofloxacin were 2.5 times more likely to have AIEC (*P* < 0.05; Supplemental Figure 1A). There was no significant association of AIEC with NOD2 and ATG16LT300A genotype in patients with CD (ICD and CCD), UC, or NI (Supplemental Figure 1, C and D). The combination of AIEC and *Enterococcus* (14% CD, 8% UC, 3% NI) was more common than AIEC and *Klebsiella*, with concurrent AIEC and *Klebsiella* absent in CD (UC 4%, NI 3%, CD 0%) (Figure 1A). AIEC strains were more frequently phylogroups B1 and D than A and B2 (*P* = 0.017) (Figure 1B and Supplemental Table 3), and they were enriched in genes encoding *lpfA* (69% AIEC; *P* = 0.0017) and *pduC* (48% AIEC; *P* = 0.0126) (Supplemental Table 3). *E*. *coli* survival in J774 macrophages and invasion of Caco-2 correlated in B1 strains (ρ = 0.593, *P* = 0.0135) but not other phylogroups (Figure 1B).

### The mucosal microbiome of AIEC shares the dysbiosis and culture of Enterococcus observed in a subset of patients with CD and ulcerative colitis.

The IBD microbiome was less ecologically diverse than the non-IBD microbiome, with lower α diversity in both UC (*P* < 0.001) and CD (*P* < 0.01) than NI (means 47, 53, and 76, respectively; Supplemental Figure 2A). The microbiomes of IBD were also more dispersed and heterogeneous than those of NI (*P* < 0.05) (Figure 1C). While many contained beneficial commensals such as *Roseburia* and *Bacteroidetes* similar to NI, a subset was distinct and highly variable enriched in opportunistic pathogens such as *Enterococcus* and *Escherichia*, including AIEC and culture of *Enterococcus* (Figure 1C). A total of 10 genera from Firmicutes (8 genera) and Bacteroidetes (2 genera) were depleted in UC and CD samples relative to NI controls (*q* < 0.2) (Figure 1D, Supplemental Figure 2B, and Supplemental Tables 5–7), with no significant difference between CD and UC, consistent with previous studies (7, 30); we did not identify an AIEC-specific microbiome. Culture of *Enterococcu*s was associated with depletion in beneficial Firmicutes and increased abundance of multiple Proteobacteria species, including *Escherichia* (Figure 1D; *q* < 0.2; Supplemental Tables 5–7 and Supplemental Figure 2C). No genera were associated with ileitis, host genotype, or culture of *Klebsiella* (Figure 1D).

### CD and ileitis shape the mucosal metabolome, with enrichment in mucosal glycerophospholipids and creatine related to AIEC.

**Figure 2:**
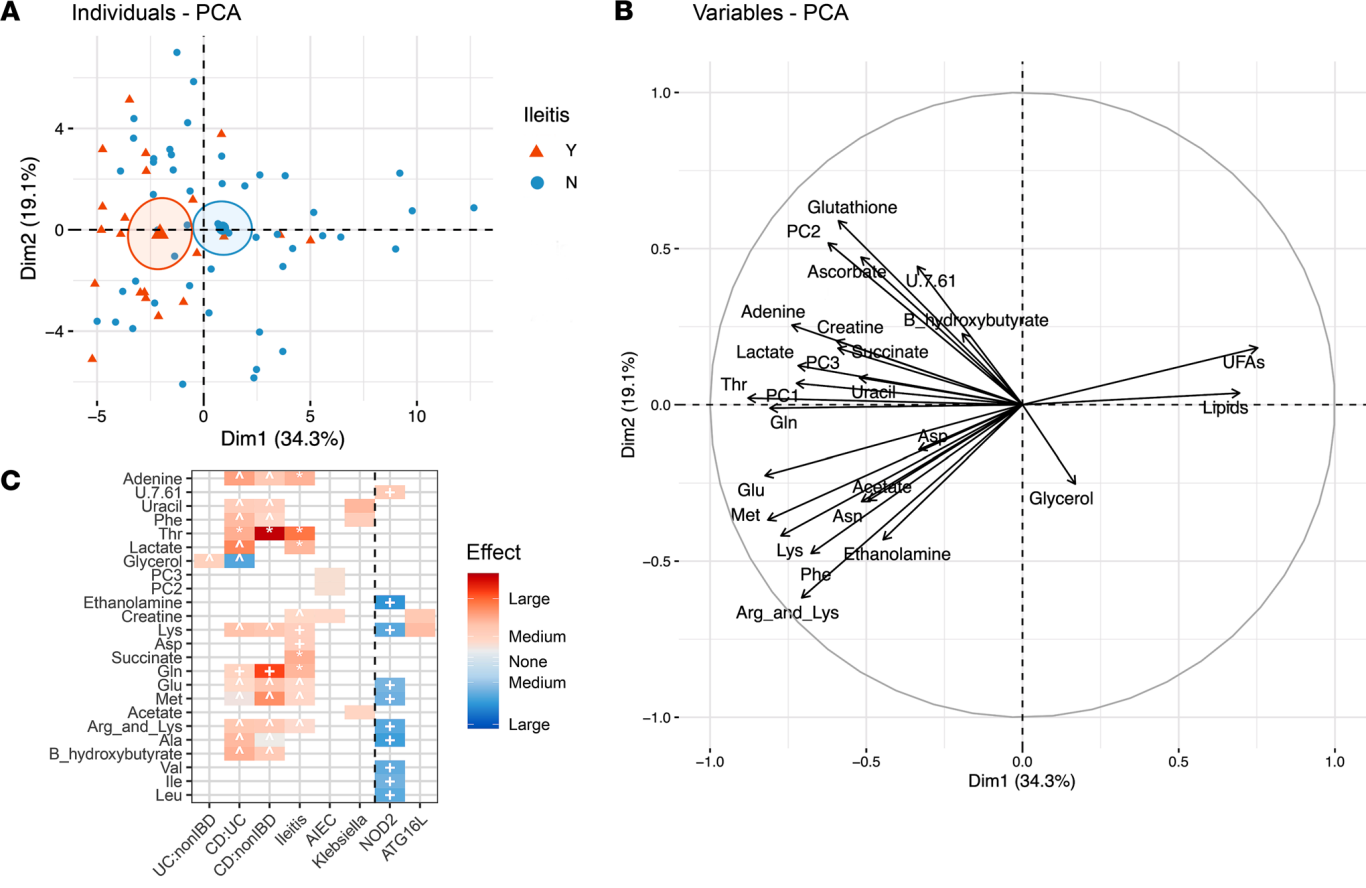
Crohn’s disease and ileitis shape the mucosal metabolome, with enrichment in glycerophospholipids and creatine related to AIEC. (**A**) Principal components analysis. Ellipses correspond to 95% confidence of data mean. (**B**) Contribution of metabolites to PCA. Arrow length corresponds to magnitude of contribution. (**C**) Metabolites associated with disease, patient genotype, and culture status. Kruskal-Wallis tests (Benjamini-Hochberg FDR) were used to determine metabolites with differential abundance between groups of interest. For all associations with *P* < 0.05, effect size (η^2^) was calculated. Intensity of color corresponds to magnitude of effect, and color corresponds to direction of effect. Symbols indicate significance of association after false discovery correction (**q < 0.01, *q < 0.05, ^+^*q* < 0.1, ^ *q* < 0.2).

In contrast to the microbiome, the metabolome segregated according to IBD and ileitis phenotypes (Figure 2A and Supplemental Figure 3A). The abundance of unsaturated fatty acids (UFAs), lipids, and glycerol was inversely correlated with the abundance of most other metabolites (Figure 2B, Supplemental Figure 3B, and Supplemental Table 8). Threonine (Thr) and glutamine (Gln) were most strongly associated with CD (*q* < 0.1), while glycerol was modestly linked to UC (*q* < 0.2) (Figure 2C and Supplemental Tables 5–7). Ileitis amplified the abundance of CD-associated metabolites, with a large effect size for Thr (η^2^ = 0.14, *q* < 0.05) and moderate effect size for adenine, lactate, succinate, and Gln (η^2^ = 0.09–0.10, *q* < 0.05) (Figure 2, B and C; Supplemental Figure 3C; and Supplemental Tables 5–7). Culture of AIEC correlated with creatine and glycerophospholipids (PC 2,3) (η^2^ = 0.05, *q* > 0.2), metabolites that cosegregate with ileitis and CD (Figure 2, B and C; Supplemental Figure 3D; and Supplemental Tables 5–7). Thr and Gln, the metabolites most strongly associated with CD and ileitis, were highly correlated with glutathione (GSH) and ascorbate (*P* = 0.48–0.83, *q* < 0.0001) and inversely correlated with lipids and UFAs (*P* = –0.23 to 0.51, *q* = 5.2 × 10^–6^ to 5.6 × 10^–2^) (Figure 2B, Supplemental Figure 3B, and Supplemental Table 8). In total, the metabolomic profile of CD, ileitis, and AIEC is consistent with mucosal inflammation, hypoxia, oxidative stress, and lipid peroxidation (31–34). *Klebsiella* was weakly associated with uracil, phenylalanine (Phe), and acetate (*q* > 0.25; Figure 2C). CD risk variants of NOD2 were inversely associated with mucosal amino acids and EA but were positive with unknown compound X 7.61, with many metabolites moving in the opposite direction to CD and ileitis (η^2^ = 0.06–0.1, *q* < 0.1; Figure 2C and Supplemental Tables 5–7). ATG16LT300GG was associated with creatine and lysine (Lys) (Figure 2C and Supplemental Tables 5–7). No metabolites were associated with *Enterococcus* culture.

### Ileal E. coli utilizes inflammation associated metabolites and glycerophospholipids for growth.

**Figure 3:**
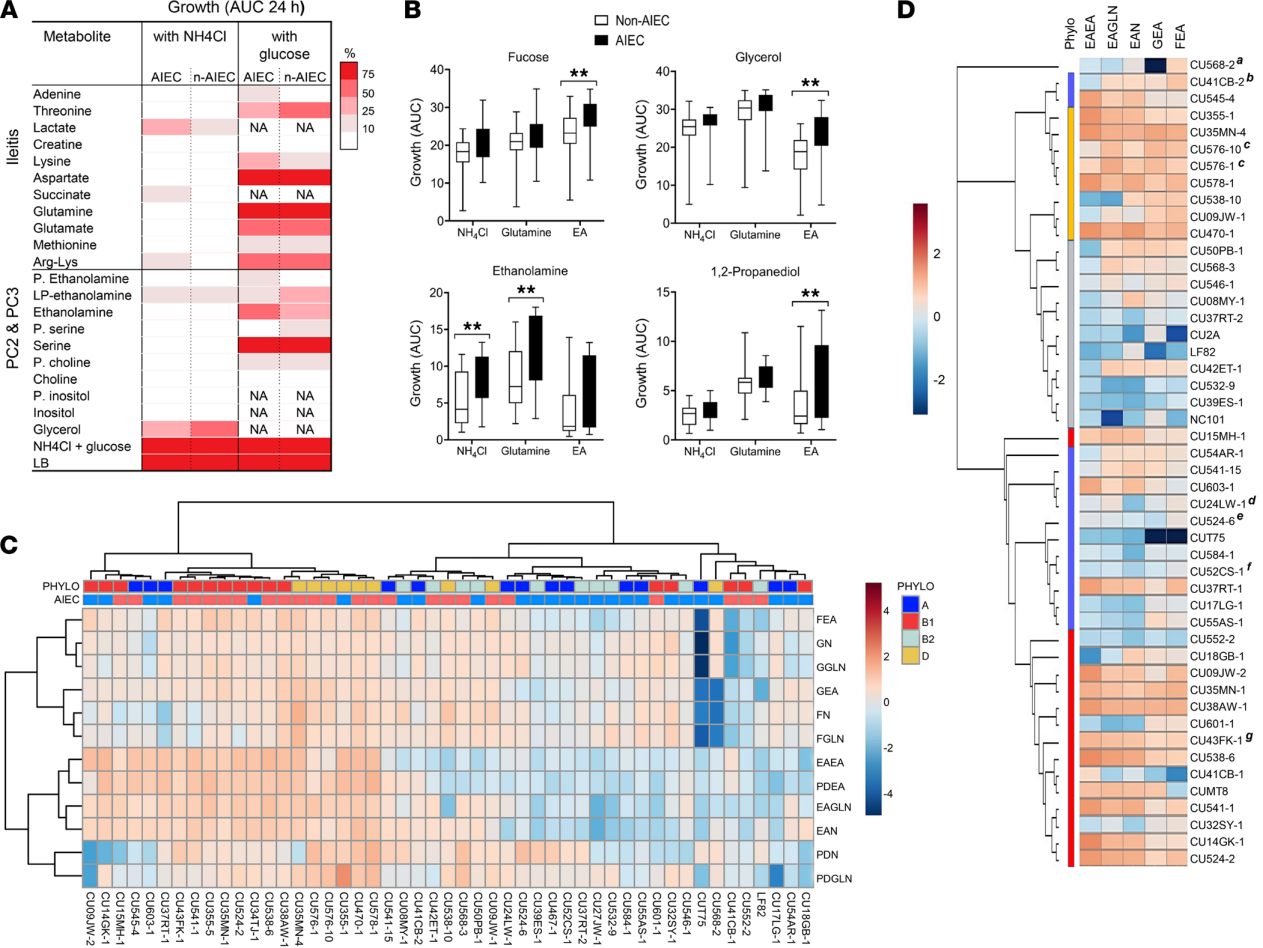
Ileal *E.* *coli* use inflammation-associated metabolites and glycerophospholipids for growth, with a differential effect of ethanolamine for AIEC. (**A**) Heatmap of growth (AUC) of ileal AIEC and non-AIEC (*n* = 10) on ileitis metabolites and constituents of ^1^H-NMR peaks PC2 and PC3. Data from 1 of 3 independent experiments (2 replicates). Multiple Mann-Whitney *U* test. (**B**) Growth (AUC ± SD) of 49 patient derived ileal *E*. *coli* (AIEC and non-AIEC) on different combinations of carbon and nitrogen. Mann-Whitney *U* test. (**C**) Heatmap of growth (AUC) of 49 ileal *E*. *coli* (AIEC red, non-AIEC blue) from phylogroups A, B1, B2, and D on combinations of fucose (F), ethanolamine (EA), glycerol (G), NH_4_Cl (N), glutamine (GLN), and 1,2-propanediol (PD). (**D**) Dendogram of the *eut* operon aligned with growth on EA. Vertical color blocks indicate phylogroup. Superscripts indicate the presence of insertions, deletions or stop codons: ^a^3 bp deletion in *eutJ*, 12 bp deletion in *eutK*, 3 bp deletion in *eutG*; ^b^23 bp insertion in *eutQ*; ^c^110 bp deletion in *eutH*; ^d^stop codon in *eutD*; ^e^insertion sequence in *eut* operon and *eutBCL* deletion; ^f^24 bp deletion in *eutB*; and ^g^stop codon in *eutR*. Substrates: fucose 20 mM; glycerol 20 mM; 1,2-propanediol 20 mM; NH_4_Cl 19 mM; ethanolamine 20 mM; Glutamine (GLN) 2.5 mM versus glucose 20 mM in M9 minimal media. (**B**–**D**) Average AUC of 3 independent experiments (2 replicates). Data are shown as mean ± SD. **P < 0.01.

To test the hypothesis that inflammation-associated metabolites support the growth of ileal *E*. *coli* and AIEC, we grew 5 AIEC, including prototypical AIEC LF82, and 5 non-AIEC spanning the A, B1, B2, and D phylogroups in media containing NH_4_Cl or glucose and ileitis-associated metabolites (Figure 3A and Supplemental Table 9). All metabolites except creatine supported growth of AIEC (Figure 3A and Supplemental Table 9), with no significant differences between AIEC and non-AIEC (Figure 3A). For carbon sources, growth of ileal *E*. *coli* on lactate, succinate, and Arg-Lys was 10%–50% of glucose (Figure 3A). For nitrogen sources, growth on Gln and Asp was > 75% of NH_4_Cl (Figure 3A), with growth of strains CU541-15 and CU578-1 on Gln and LF82 on Asp (Supplemental Table 9) exceeding NH_4_Cl, which is considered an optimal nitrogen source for *E*. *coli* (35). Growth on Thr, Lys, glutamate (Glu), and Arg-Lys ranged from 10% to 75% of NH_4_Cl (Figure 3A and Supplemental Table 9).

We next sought to determine whether metabolites associated with ^1^H-NMR peaks PC2 and PC3 support *E*. *coli* growth. PC2 and PC3 are composed of glycerol and phosphatides, such as phosphatidylcholine (PC), phosphatidylethanolamine (PE), phosphatidylserine (PS), and phosphatidylinositol (PI) and can be degraded to lysophophatidylethanolamine (LPE), EA, serine (Ser), choline, and inositol (36, 37). Ileal *E*. *coli*, with the exception of non–AIEC T75, were unable to utilize choline and inositol for growth in media containing NH_4_Cl or glucose (Figure 3A and Supplemental Table 9). Growth on Ser as nitrogen was > 75% of growth with NH_4_Cl (Figure 3A), consistent with previous studies linking L-serine metabolism to the fitness of AIEC in the inflamed intestine (38). LPE served as a source of carbon and nitrogen, supporting up to 50% of growth on NH_4_Cl for non-AIEC (Figure 3A) (37). Growth on PE, PS, PC, and PI was < 25% of glucose plus NH_4_Cl on average (Figure 3A), consistent with the need for cleavage of these moieties by intestinal phospholipase (37, 39, 40).

We observed substantial variation in the ability of individual AIEC and non-AIEC strains to use EA as an N source, with growth of AIEC LF82 much lower than other AIEC, and some non-AIEC (39EJS-1, 568-2; Supplemental Table 9). The ability of AIEC and non-AIEC to utilize EA as a source of nitrogen is in agreement with previous reports (21, 23, 28, 29). As a carbon source, EA did not promote significant growth in M9 media over 24 hours. However, when growth was extended for 48–72 hours, 2 strains were weakly adapted to use EA as a C source (Supplemental Figure 4A), and this effect was enhanced when *E*. *coli* were inoculated directly into M9 after overnight growth in LB without washing (Supplemental Figure 4B).

Substrate use by murine AIEC (CUMT8, NC101) and *pduC*^+^ Th17-inducing AIEC CU2A (15, 19) aligned with ileal AIEC in the same phylogroup. The effect of glycerophospholipids (PC, PI, PS, choline) and ileal metabolites (adenine, aspartate, glycerol, lactate, and creatine) on growth relative to NH_4_Cl or glucose was highest for strains that grew poorly on NH_4_Cl and glucose compared with LB (LF82, T75, NC101). However, growth of these strains remained substantially lower than other AIEC and non-AIEC (Supplemental Table 9).

### EA and Gln enhance the growth of ileal E. coli on nonglucose carbon sources, with a differential effect of EA on AIEC.

On the basis of these results, we examined the ability of 49 *E*. *coli* strains isolated from the ileal mucosa of our patient cohort (24 AIEC and 25 non-AIEC) to utilize combinations of carbon and nitrogen associated with a healthy gut (fucose, glycerol, NH_4_Cl), ileitis (Gln), PC2, PC3 (EA), and 1,2-PD, a product of fucose metabolism linked to the AIEC pathotype (*pduC*) and Th17-mediated intestinal inflammation (15). We selected Gln because of its effect size (Figure 2C), ability to support growth of ileal *E*. *coli* (Figure 3A), and functional importance in the small intestine, and we selected EA because of its impact on growth, potential to differentially support AIEC, and to gain insights into the cometabolism of EA, fucose, and 1,2-PD that involves paralogous vitamin B_12_-dependent MCP (20). As a nitrogen source, Gln supported higher growth of AIEC and non-AIEC on glycerol and EA than NH_4_Cl and EA (Figure 3, B and C), whereas EA supported higher growth on fucose and 1,2-PD (Figure 3, B and C). AIEC and non-AIEC grew similarly on glucose, glycerol, and fucose (Figure 3, B and C), but AIEC were better able to utilize EA as a source of nitrogen and carbon than non-AIEC (*P* < 0.05; Figure 3, B and C). Growth on EA varied by phylogroup, with B1 and D the most and B2 the least able to use EA as a source of carbon (+NH_4_Cl) and nitrogen (+EA) (*P* < 0.05) (Figure 3C and Supplemental Figure 4, C–F). *E*. *coli* strains containing the *pduC* and *lpfA* virulence genes were better able to utilize EA (*P* < 0.05), with increased growth of *pduC* strains on EA and fucose and increased growth of *lpfA* strains on EA with fucose or glycerol (*P* < 0.05) (Supplemental Figure 4, G and H). The ability of AIEC and non-AIEC to utilize EA alone, or in combination with glycerol, NH_4_Cl, and Gln, correlated with the invasion of epithelial cells (*P* < 0.05), whereas growth on 1,2-PD and NH_4_Cl correlated with replication in macrophages (*P* < 0.05; Supplemental Table 10 and Supplemental Figure 5, A and B).

To investigate variation in EA use, we aligned the *eut* operon of 48 *E*. *coli* strains (26 AIEC and 22 non-AIEC). We identified segregation and clustering by phylogroup (Figure 3D and Supplemental Figure 5C). Deletions or insertions were present in 6 strains, including 2 AIEC strains (CU576-1 and CU576-10), with deletions in *eutK*, e*utQ*, and *eutB* associated with the inability to utilize EA as a source of C and N (Figure 3D). A 110 bp deletion in *eutH* in AIEC CU576-1 and CU576-10 had a minimal effect on growth on EA. We did not identify insertions or deletions in B2 strains— e.g., AIEC LF82, CU2A, and NC101 — that grew poorly on EA as the sole source of C and N (Figure 3D, Supplemental Figure 4, E and F, and Supplemental Figure 5C). The low ability of B2 strains to grow on EA could reflect phylogroup-specific polymorphisms in the *eut* operon or, alternatively, the need for an electron acceptor such as tetrathionate (TTH) or thiosulphate (TS) to utilize EA as a C source described for *Salmonella* and AIEC LF82 (5, 21, 41). The growth of LF82 on glycerol and EA was enhanced by TTH and TS, although growth was poor relative to CU541-1 (Supplemental Figure 5D). However, TTH and TS did not augment the growth of B1 AIEC CU541-1 under the same conditions (Supplemental Figure 5D). Thus, the cause of differences in EA metabolism and requirement for TTH and TS between AIEC phylogroups remain to be determined.

### Genes related to metabolism, stress, biofilm formation, motility, chemotaxis, and sensing in AIEC are differentially regulated by EA, Gln, glycerol, and fucose.

**Figure 4:**
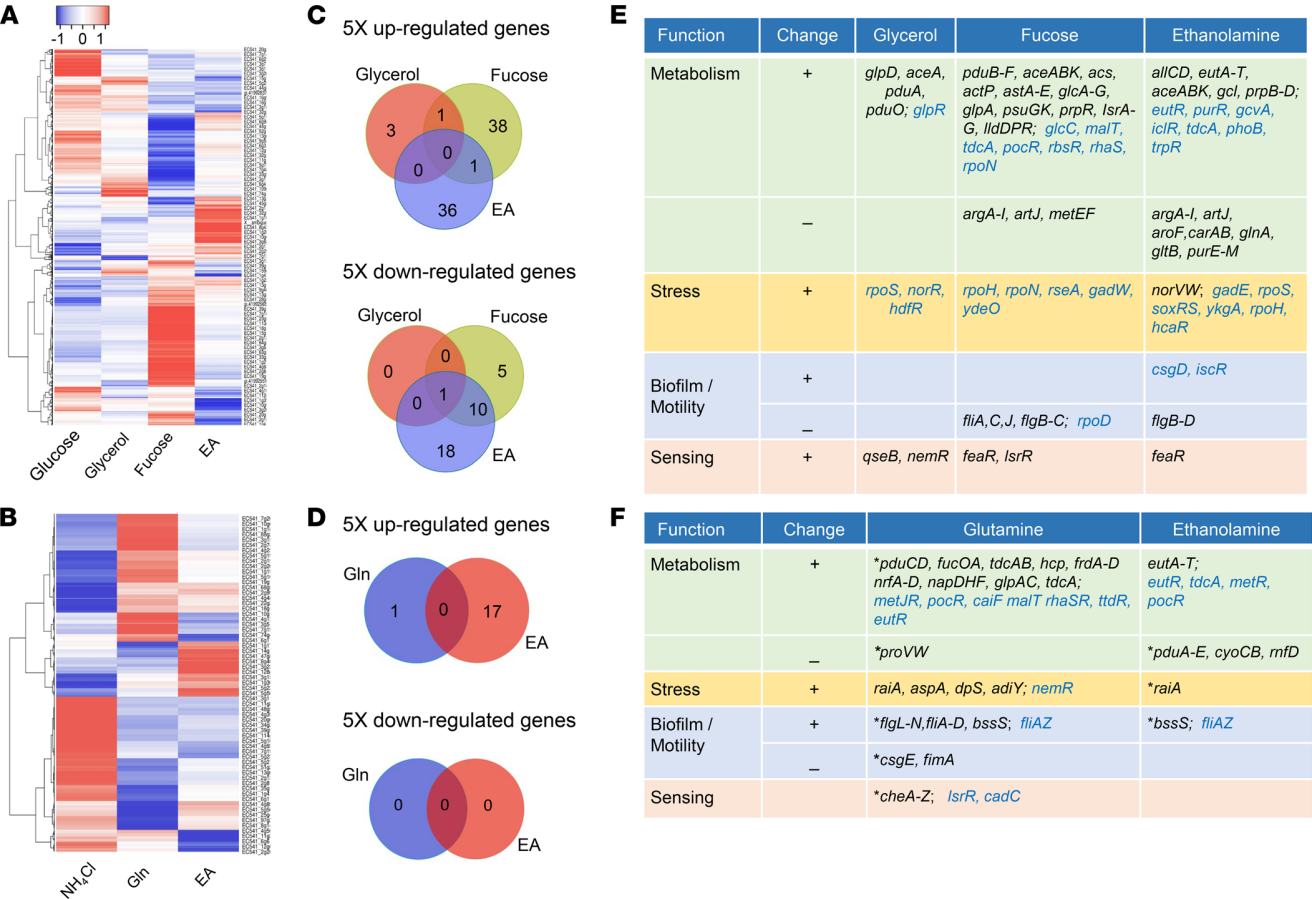
Transcription of genes related to metabolism, stress, biofilm, motility, chemotaxis, and sensing in AIEC is differentially regulated by ethanolamine, glutamine, glycerol, and fucose. (**A** and **B**) Transcriptional profiles for AIEC CU541-1 grown in mucosal sources of carbon (**A**) and nitrogen (**B**). (**C** and **D**) Differential abundance (5-fold cutoff, *P* ≤ 0.05) of transcribed genes by carbon (**C**) and nitrogen sources (**D**). Venn diagrams show differential expression calculated using edgeR (65) and cuffdiff (66). (**E** and **F**) Differentially abundant genes and transcription factors (in bold) by carbon (**E**) and nitrogen (**F**) sources. For nitrogen sources (**F**), genes with 2-fold cutoff are indicated by an asterisk.

To delineate substrate-responsive pathways in AIEC, we analyzed the transcriptomes of ileal CD AIEC CU541-1 (B1, *lpfA^+^*, *pduC^+^*, *eut^+^*) grown in microaerophilic conditions (5% O_2_, 5% CO_2_) simulating the small intestine (42), with mucosal sources of carbon (glucose, fucose, glycerol, EA) and nitrogen (NH_4_Cl, Gln, EA). There was substantial overlap in transcriptional profiles for glucose and glycerol, but there were individually distinct profiles for fucose and EA as carbon sources (Figure 4A) and for NH_4_Cl, Gln, and EA as nitrogen sources (Figure 4B). Analysis by gene ontology highlighted differential transcription by carbon source for amino acids, protein, carbon, biofilm formation, and chaperone (Supplemental Figure 6A). Those same functional groups plus motility were impacted by the N source, with a greater effect of Gln than EA, except for amino acids (Supplemental Figure 6B). Five-fold differences in transcript abundance for carbon sources relative to glucose were dominated by fucose and EA (Figure 4C and Supplemental Tables 11 and 12). Nitrogen source had less impact, with 5-fold upregulation restricted to 17 genes in EA and 1 with Gln (Figure 4D and Supplemental Table 13). Integration of gene transcription for carbon (5-fold) and nitrogen (2-fold; Supplemental Figure 6, C and D, and Supplemental Tables 14 and 15) sources with transcription factors highlighted differences in metabolism, stress, biofilm/motility, and sensing (Figure 4, E and F, and Supplemental Tables 16–20). Growth on EA as a carbon source was dominated by increased transcription of genes and transcription factors involved in the *eut* operon, glyoxylate shunt (*aceABK*, *iclR*), and methylcitrate cycle (*prpB–D*), with concurrent anaerobic degradation of Thr to Gly and Ser (*tdcA*, *gcvA*), phosphate regulation (*phoB*), and the repressor of purine nucleotide biosynthesis (*purR*) (Supplemental Table 16). Pathways related to the synthesis of Gln and Glu (*glnA*, *gltB*), purine (*purE–M*), and Arg (*argA–I*) were downregulated (Figure 4E and Supplemental Table 16). Growth in EA was accompanied by a pleiotropic response to stress in general (*rpoS*), acid (*gadE*), heat (*rpoH*), superoxide (*soxS*, *soxR*, *ykgA*), nitrosative (*norV*, *soxS*), and oxidative stress (*soxS*, *hcAR*) (Figure 4E).

There was also upregulation of factors associated with biofilms (*csgD*, *iscR)* and epinephrine sensing (*feaR*) (Figure 4E). Growth in fucose was associated with upregulation of genes and transcription factors associated with metabolism of 1,2-PD (*pduB–F*, *pocR*), acetyl-CoA (*acs*), glycolate to glyoxylate to malate (*glcA–G*), L-lactate metabolism (*llDPR*), propionate (*prpR*), Arg (*astA–E*, *rpoN*), and degradation of Thr (*tdcA*), with downregulation of *argA–I* (Figure 4E). Transcription factors associated with acid stress (*gadW*, *ydeO*), heat shock (*rpoH*), and sensing of epinephrine (*feaR*) were also upregulated, whereas those associated with motility (*fliACJ*, *metEF*, and *rpoD)* were downregulated (Figure 4E). Growth in glycerol upregulated pathways directly related to glycerol metabolism (*glpD*, *glpR*) and the glyoxylate shunt (*aceA*). It was also accompanied by upregulation of genes involved in propanediol uptake and metabolism (*pduA*, *pduO*), stress (*rpoS*, *norR*, *hdfR*), and sensing (*qseB*, *nemR*) (Figure 4E).

The transcriptional response to different nitrogen sources was limited to > 5-fold upregulation of the *eut* operon with EA (Figure 4D and Supplemental Table 13). Growth on EA and Gln induced a concordant increase in transcription factors *eutR*, *tdcA*, and *metR* (Figure 4F). Concurrent transcription of *cadC* and *tdcA* with Gln links bile salt and acid sensing (*cadC*) with induction of anaerobic use of Thr (*tdcA)* (43). Surprisingly, EA and Gln induced *pocR*, the regulator of the *pdu/cob* operon (Figure 4F), and there was downregulation of *pduA–E* with EA and upregulation of *pduCD* and *fucOA* with Gln (Figure 4F), supporting crosstalk between Gln and EA in the metabolism of fucose and 1,2-PD. In contrast to carbon sources, Gln and EA induced operons related to motility (*flg/fli*) and biofilm (*bssS*) formation, with concordant upregulation of chemotaxis-associated genes with Gln (*cheA–Z*) (Figure 4F).

Many of the stress-associated genes and transcription factors induced by EA and fucose are implicated in the ability of enteropathogens to resist nitrosative, oxidative, and superoxide stress in the inflamed intestine (5, 6, 44). Gene *feaR* is involved in epinephrine sensing and chemotaxis of EHEC (45), and the transcriptional factor *tdcA* has been linked to growth of AIEC in the inflamed intestine (38) and bile acid sensing via *cadC* (43). Sequences for *norV*, *soxR*, *gadE*, *rpoH*, and *tdcA* were present in all strains in our pool of patient derived AIEC and non-AIEC, and *cadC* and *ykgA* were present in all but 6 strains, predominantly phylogroup A, non-AIEC (Supplemental Table 3). *FeaR* and *hcaR* were absent in B2 strains, with similar distributions in AIEC and non-AIEC (Supplemental Table 3*)*.

### Cometabolism of amino acids and GSH enhances the growth of AIEC on EA.

**Figure 5:**
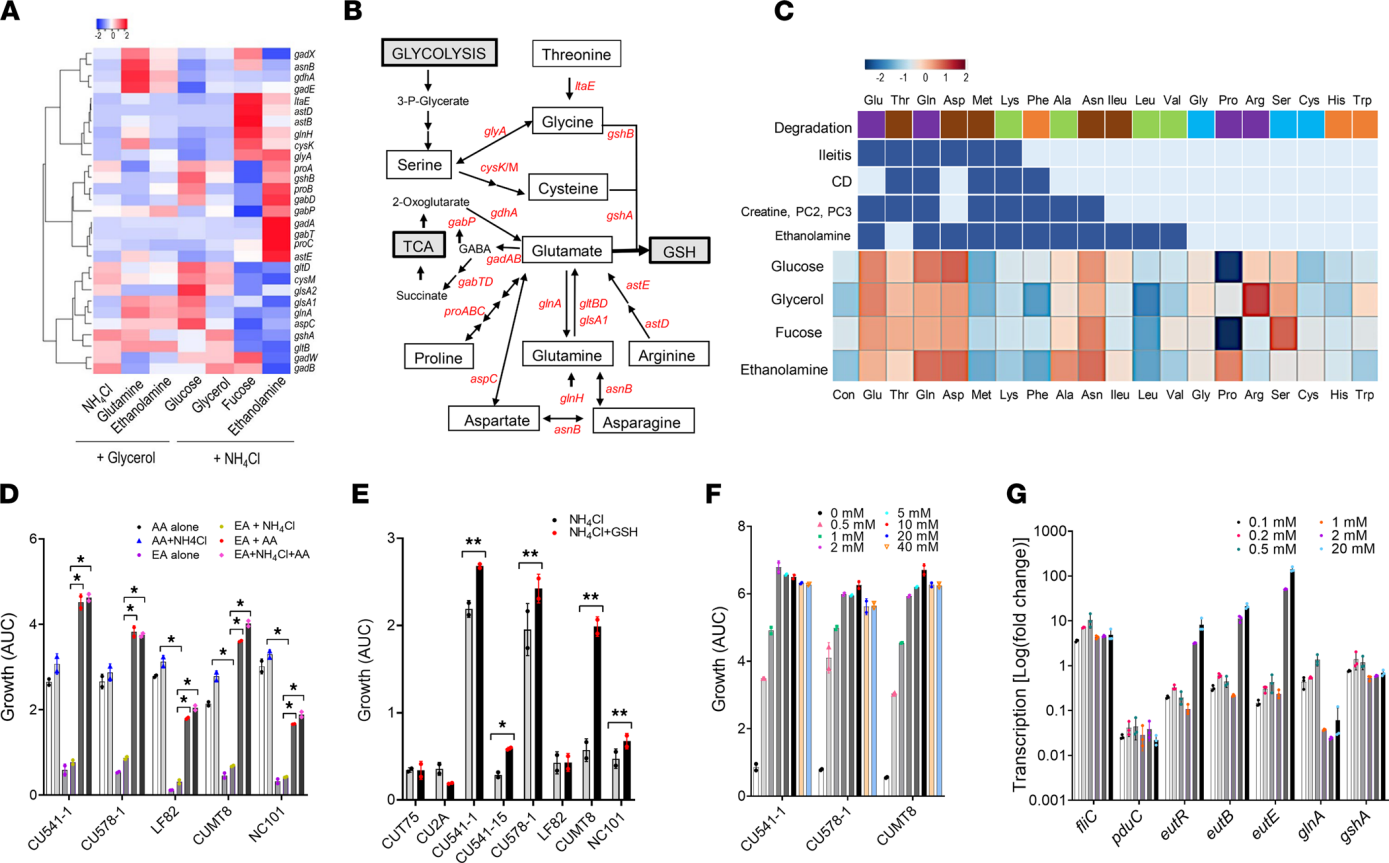
Cometabolism of amino acids modulates AIEC growth on ethanolamine, fucose, and glycerol, with GSH augmenting growth on ethanolamine. (**A**) The transcriptional profiles for CU541-1 related to amino acid metabolism and the γ-glutamyl cycle. (**B**) Pathways and genes involved in amino acid catabolism and γ-glutamyl cycle. (**C**) Heatmap of the effect of exogenous single amino acids (10 mM) on growth of AIEC CU541-1 (CFU) in mucosal carbon sources. Con, control. Carbon source + NH_4_Cl. Amino acids are grouped by their degradation pathways (46) (indicated by color) and differential abundance in the ileal metabolome (dark blue). Data are shown from 1 of 3 independent experiments (2 replicates). (**D**) Effect of mixed amino acids (Glu, Thr, Gln, Asp, Asn, and Pro; 10 mM) on AIEC growth in M9 + 20 mM EA. (**E**) Effect of GSH (2.5 mM) on growth of AIEC and non-AIEC (CUT75) in M9 + EA + NH_4_Cl. (**F**) Impact of EA (0–40 mM) on growth of AIEC in fucose (20 mM) in M9 media. (**G**) Transcript abundance during growth of AIEC CU541-1 on fucose and EA (0–20 mM). (**D**–**G**) Data from 1 of 3 independent experiments (2 replicates). Two-way ANOVA with Tukey’s multiple-comparison test. Data are shown as mean ± SD. **P < 0.01, *P < 0.05.

The ability of ileal *E*. *coli* and AIEC to utilize mucosal amino acids for growth (Figure 3A), the effect of Gln on EA use (Figure 3B), and the differential abundance of transcripts related to amino acid biosynthesis and degradation on different sources of carbon and nitrogen (Supplemental Figure 6, A and B, and Supplemental Figure 7, A–D) prompted an in-depth analysis of amino acid metabolism in AIEC CU541-1 during growth on EA. The transcriptional profile of CU541-1 with EA and NH_4_Cl (Figure 5A and Supplemental Table 21) was characterized by upregulation of Gln transport (*glnH*) and degradation of Thr and Ser to Gly; Arg, Asp, and Pro to Glu; and Gly and Glu to GSH (Figure 5, A and B). There was a marked increase in GABA permease (*gabP*) and GABA synthesis from Glu (*gadA*), directed toward succinate (*gabTD*) and the TCA cycle (Figure 5, A and B). This transcriptional profile supports a multifaceted role of the metabolism of endogenous amino acids in protection against oxidative and acid stress, as well as energy during use of EA as a source of carbon and nitrogen (46).

To test the ability of exogenous amino acids to augment growth on EA, we grew AIEC CU541-1 on a panel of 19 amino acids that included those with differential abundance in ileitis, CD, and AIEC mucosa and those cocorrelated with AIEC (creatine, PC2, PC3) and EA (Figure 3A and Supplemental Figure 3B). Ileitis-associated Gln, Glu, Thr, and Asp and regionally available Ser, Ala, Asn, and Gly enhanced the growth of AIEC CU541-1 on glucose, glycerol, fucose, and EA (Figure 5C and Supplemental Figure 7E). Growth on glucose, glycerol, fucose, and EA was highest with Asp, Arg, Ser, and Asp, respectively. Additionally, we observed that suppression of growth in glucose, glycerol, fucose, and EA was greatest with Pro, leucine, Pro, and Phe, respectively (Figure 5C and Supplemental Figure 7E). Differences in the ability of exogenous amino acids to augment growth were most apparent for Pro and Ala for EA, Arg for glycerol, and Ser for fucose (Figure 5C). The differential impact of Pro and Phe on growth in EA (Figure 5C and Supplemental Figure 7E) was maintained in other AIECs (CUMT8, CU578-1, CU18GB-1; Supplemental Figure 7F) and was accompanied by upregulation of *proC*, *-P*, *-V*, and -*Y* and by downregulation of *proW* relative to glucose (Supplemental Figure 7G). To model the effect of the ileal mucosal microenvironment on the growth of AIEC, we evaluated the ability of a mixture of differentially abundant and regionally available amino acids (Figure 5C) to promote growth of AIEC on EA. Mixed amino acids had a synergistic effect on growth of AIEC CU541-1, CU578-1, and CUMT8 on EA, but not LF82 and NC101, compared with NH_4_Cl + amino acids (Figure 5D).

The correlation of mucosal Gln and GSH with AIEC in the mucosal metabolome, the ability of Gln to enhance growth of AIEC in EA (Figure 3A), and the abundance of pathways related to metabolic stress and the γ-glutamyl cycle suggest that GSH may influence the use of EA by AIEC. We found that GSH augmented the growth of 5 of 7 AIEC (Figure 5E) on EA + NH_4_Cl and EA alone (Supplemental Figure 7H), with the exception of B2 strains that grew poorly on EA (LF82 and CU2A). The addition of GSH during growth in EA + NH_4_Cl + Gln did not further enhance growth (Supplemental Figure 7I). Collectively, these findings point to a role of exogenous and endogenous amino acids and GSH in supporting the growth of AIEC on EA.

### EA acts as a signal and substrate during growth on fucose.

Our finding that EA was the optimal nitrogen source for fucose and 1,2-PD was unexpected, as cometabolism of these substrates in *Salmonella* is regulated to avoid detrimental mixing of components of 2 MCPs, with the use of 1,2-PD preferred over EA (47). The transcriptional changes in *pocR*, *eutR*, *pduCD*, and *fucOA* with Gln, and reciprocal changes in the transcription of *pocR* and *pduA–E* with EA (Figure 4, E and F) point to crosstalk between the *eut* and *pdu* MCP and the γ-glutamyl cycle. To investigate this interaction, and to establish the minimal concentration of EA for growth on fucose, we grew AIEC (CU541-1, CUMT8 and CU578-1) in M9 media with fucose (20 mM) and EA (0-20 mM). We found that EA stimulated growth in fucose at 0.5 mM with a maximal effect at 2–5 mM, and it reduced growth at 10–20 mM (Figure 5F). Transcriptional analysis revealed decreased *pduC* transcription relative to fucose alone at EA concentrations of 0.1–20 mM (Figure 5G). Conversely, *fliC* transcription was upregulated (Figure 5G). Changes in *pduC* and *fliC* transcription at EA concentrations <2 mM suggest that this effect is independent of EA metabolism, indicated by upregulation of *eutRBE* (2 and 20 mM) (Figure 5G). Transcription of γ-glutamyl cycle–related *glnA* and *gshA* was highest at 0.1–0.5 mM EA, and it was repressed at 1–20 mM, preceding increased transcription of the *eut* operon. These findings support a concentration-dependent effect of EA, with optimal growth on fucose as a carbon source and EA as a nitrogen source at concentrations of 2 mM, the concentration in healthy bovine small intestine (48), and regulatory effects (e.g., *pduC* and *fliC)* at lower concentrations.

### EA and Gln enhance AIEC motility, infectivity, and proinflammatory responses in vitro.

**Figure 6:**
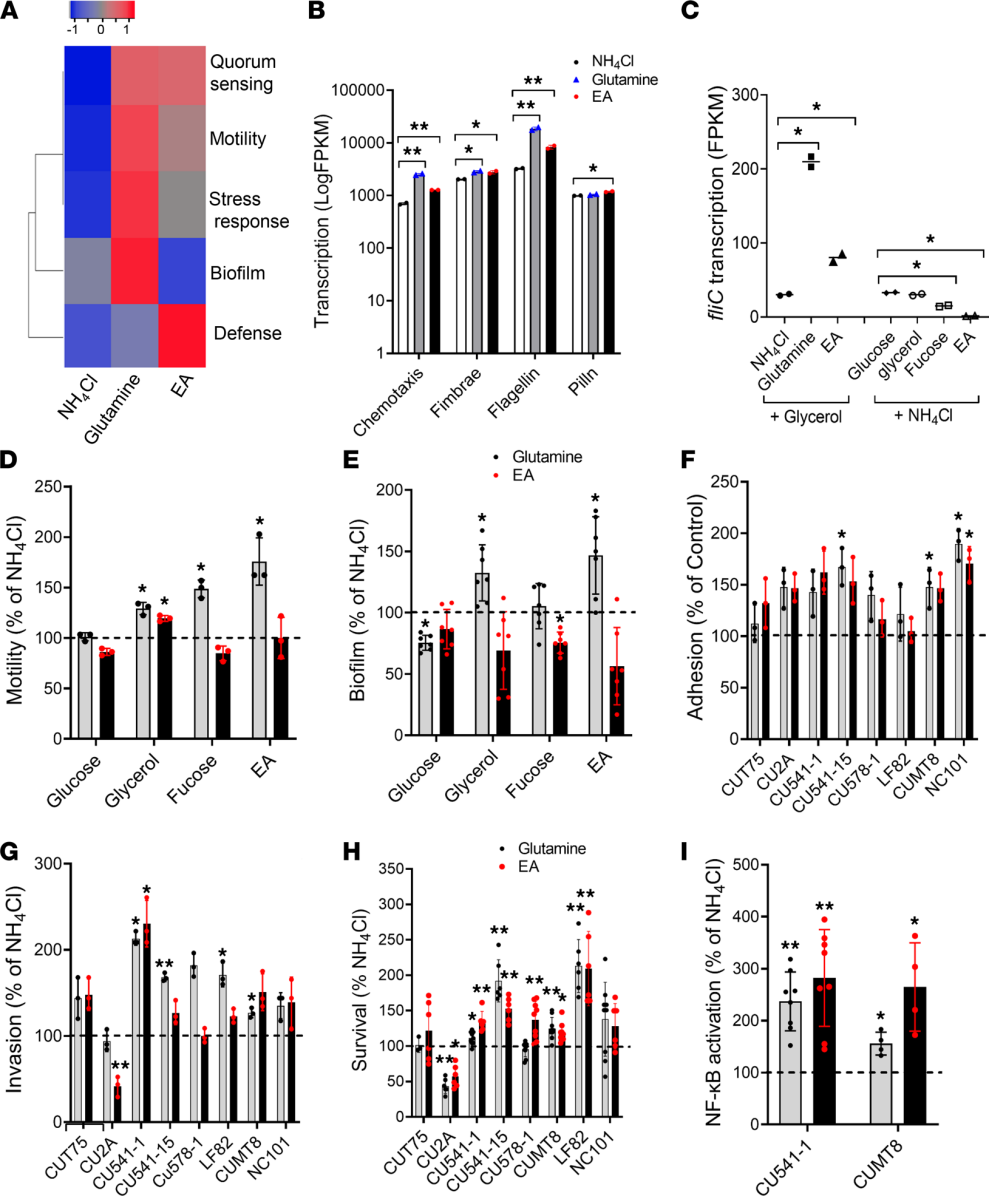
Ethanolamine and glutamine enhance AIEC motility, infectivity and proinflammatory responses in vitro. (**A**) Transcriptional analysis of virulence gene expression in AIEC CU541-1 by nitrogen source. (**B** and **C**) Abundance of transcripts in pathways associated with chemotaxis, motility, and adhesion (Mann-Whitney *U* test) (**B**) and with *fliC* (**C**) (2-way ANOVA with Tukey’s multiple-comparison test). (**D**–**I**) Functional evaluation of the effects of ethanolamine (EA) and glutamine on motility, biofilm, adhesion, invasion, replication in macrophages, and NF-κB activation. Related methods are described in the Methods and Supplemental Methods. (**D**–**I**) At least 3 independent experiments were performed for each assay (3 replicates). Two-way ANOVA with Dunnett’s multiple-comparison test. Data are shown as mean ± SD. **P < 0.01, *P < 0.05.

In contrast to the impact of carbon source on metabolism, nitrogen source — especially Gln and EA — had the greatest impact on the transcription of genes associated with chemotaxis, fimbriae, flagella, and pili, related to virulence (Figure 6, A–C, and Supplemental Tables 22 and 23). To assess functionality, we tested the effect of Gln and EA on motility of AIEC CU541-1 with different carbon sources (Figure 6D). Gln increased motility on glycerol, fucose, and EA, with EA enhancing motility on glycerol (Figure 6D). This effect was maintained in AIEC LF82 and CUMT8 (Supplemental Figure 8, A–C) and was induced by EA at 1–10 mM (CU541-1, LF82, CUMT8). Biofilm formation was enhanced by Gln in glycerol and EA, but it was reduced in glucose and fucose (Figure 6E).

We next determined the effect of Gln and EA on the AIEC pathotype with a group of 7 AIEC strains spanning the A–D phylogroups, including *pduC*^+^, Th17-inducing AIEC CU2A, murine AIEC (CUMT8, NC101), and non-AIEC (T75) (15, 19). The effect of Gln and EA on adhesion varied by strain, enhancing adhesion for 3 of 7 and 1 of 7 AIECs, respectively (Figure 6F). Gln enhanced invasion by 5 of 7 AIEC compared with only 1 of 7 AIEC with EA (Figure 6G), in line with the differential impact of Gln on motility (Figure 6D) (24, 49). EA and Gln enhanced persistence in macrophages for 5 and 4 of 7 AIEC strains, respectively (Figure 6H). AIEC CU2A (B2, *pduC^+^*, CD-SpA feces) was the exception, with EA and Gln reducing invasion and persistence. Gln and EA enhanced NF-κB activation by AIECs in cultured epithelial cells (Figure 6I). These findings link mucosal metabolites to virulence, with EA and Gln enhancing AIEC motility, infectivity, and proinflammatory responses in vitro.

### The ability of AIEC to metabolize EA and fucose/rhamnose modulates intestinal inflammation in IBD-susceptible IL10^–/–^ mice.

**Figure 7:**
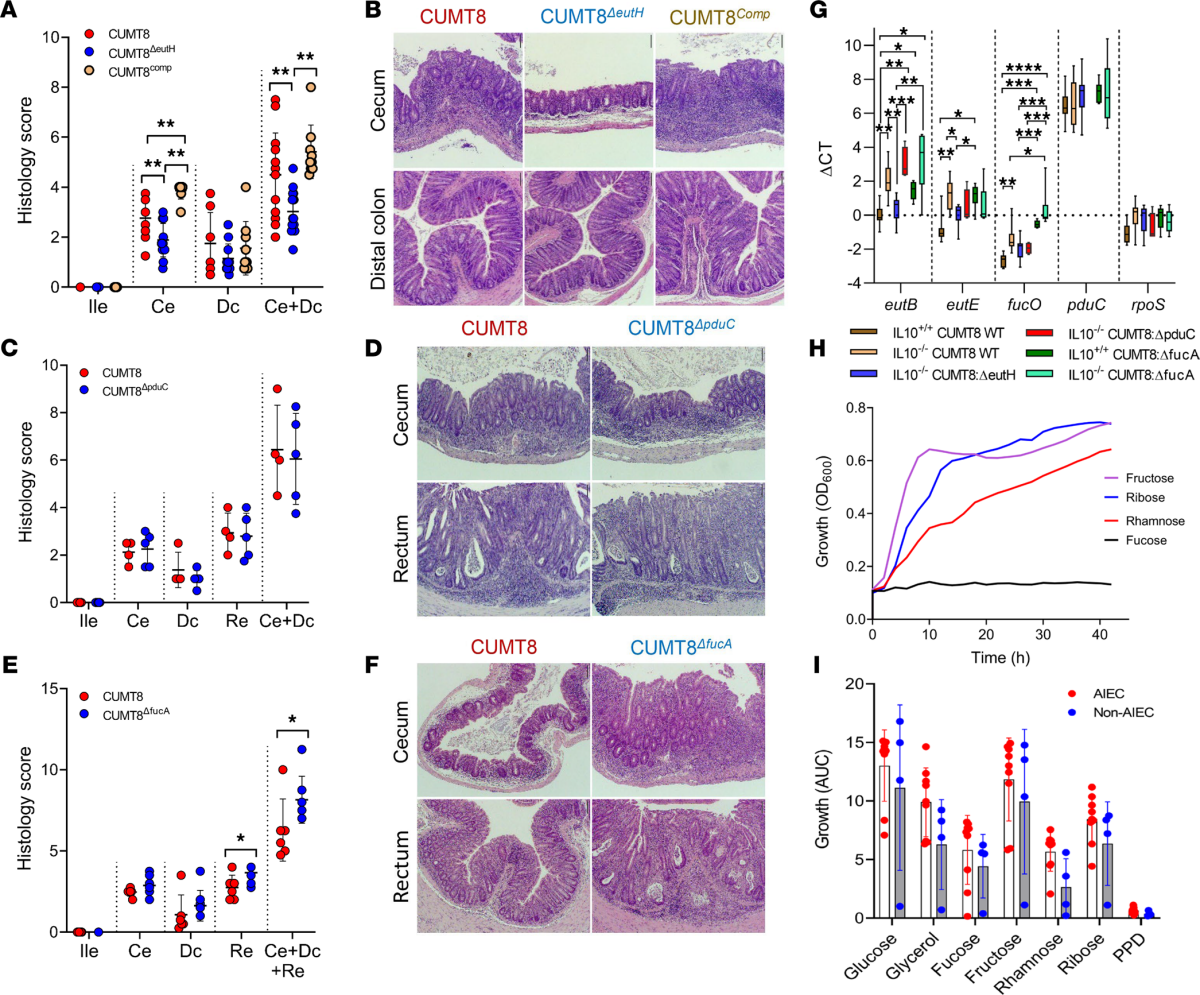
The ability of AIEC to metabolize ethanolamine and fucose/rhamnose modulates intestinal inflammation in IBD susceptible IL10^−/−^ mice. (**A**–**F**) Blinded histology scores and representative images of IBD susceptible 129SvEv IL10^−/−^ mice monoassociated with parental CUMT8 WT and derivatives (Δ*eutH*, Δ*pduC*, and Δ*fucA*). (**A** and **B**) CUMT8-WT (*n* = 15); CUMT8^ΔeutH^ (*n* = 15); complement Δ*eutH* CUMT8^comp^ (*n* = 9). (**C** and **D**) CUMT8-WT (*n* = 4); CUMT8^ΔpduC^ (*n* = 5). (**E** and **F**) CUMT8-WT (*n* = 6); CUMT8^ΔfucA^ (*n* = 8). (**G**) Transcriptional analysis of cecal contents of CUMT8 monoassociated IL10^−/−^ and IL10^+/+^ mice for genes related to ethanolamine (*eutB*, *eutE*), fucose metabolism (*fucO*, *pduC*), and stress (*rpoS*). (**H** and **I**) Sugar use Δ*fucA* (**H**) and growth (**I**) of ileal *E*. *coli* on alternate carbon sources. Ile, ileum; Ce, cecum; Dc, distal colon; Re, rectum. Symbols indicate significance of association after 1-way ANOVA Holm-Sidak’s multiple-comparison test (**A**, **C**, and **E**), or Kruskal-Wallis test with Dunn’s multiple comparison (**G**). **P < 0.01, *P < 0.05.

On the basis of our findings for EA, and previous studies of the fucose-1,2-PD pathway (14, 15, 18), we hypothesized that compromising the ability of AIEC to utilize EA, fucose, and 1,2-PD would reduce intestinal inflammation in IBD-susceptible mice. To test this hypothesis, we monoassociated IBD-susceptible 129SvEv *IL10^–/–^* and IBD-resistant *IL10^+/+^* mice with AIEC CUMT8 (WT; B1, *lpfA*^+^, *pduC*^+^) (18), a strain that induces inflammation and Th17 responses in *IL10^–/–^* mice (14, 15); clusters with AIEC CU541-1 by genotype (19) and phenotype (Figures 3, 5, and 6); and parental derivatives of AIEC CUMT8 with engineered deletions in *fucA* (Δ*fucA*), *pduC* (Δ*pduC*), and *eutH* (Δ*eutH*) (15, 19) (Supplemental Figure 8, D–H). *IL10^–/–^* mice colonized with CUMT8^ΔeutH^ developed less severe intestinal inflammation and reduced proinflammatory cytokine transcription (IFN-γ, IL-17α, and IL-12β) compared with CUMT8-WT mice (Figure 7, A and B, and Supplemental Figure 9A). *IL10^+/+^* mice did not develop inflammation with CUMT8, consistent with the role of AIEC as an opportunistic pathosymbiont (Supplemental Figure 9, D–G). Complementation of *eutH* restored the ability of CUMT8^ΔeutH^ to induce inflammation, confirming specificity for EA (Figure 7, A and B, and Supplemental Figure 9A). In contrast, *IL10^–/–^* mice colonized with CUMT8^ΔpduC^ developed a similar degree of mucosal inflammation and proinflammatory cytokine transcription to CUMT8-WT mice (Figure 7, C and D, and Supplemental Figure 9B), while those colonized with CUMT8^ΔfucA^ developed more severe inflammation and increased colonic transcription of IL-12β than CUMT8-WT mice (Figure 7, E and F, and Supplemental Figure 9C).

We note that the use of fucose by *E*. *coli* in monocolonized germ-free mice is controversial, with some studies describing transcription of *fucAO* in mucus and luminal contents and fucose-derived 1,2-PD–mediated intestinal inflammation by AIEC, while others describe a requirement for cocolonization with mucolytic *B*. *thetaiotaomicron* to liberate fucose (4, 15, 29). To gain further insights into the interaction between the *pdu* and *eut* MCP during colonization, we performed transcriptional analysis of the cecal contents of CUMT8 monoassociated *IL10^–/–^* and *IL10^+/+^* mice. The transcription of *eut* genes in cecal contents was higher in the uninflamed intestine of *IL10^+/+^* mice than inflamed *IL10^–/–^* mice (Figure 7G), confirming the use of EA in the healthy gut and the need for host-susceptibility in the inflammatory process (28, 29).

The transcription of *fucA*, *fucO*, *pduC*, *eutBCHR*, Gln (*gltB*), GSH (*gshB*) and stress (*rpoS*, *uidA*) supports the cometabolism of EA and fucose/rhamnose by AIEC CUMT8 in vivo (Figure 7G and Supplemental Figure 10A). The transcription of *pduC* was uniformly low in all groups of mice, despite variable transcription of *fucA* and *fucO*, reduced transcription of *fucO* in Δ*fucA*, and the ability of fucose to induce transcription of *fucA*, *fucO*, and *pduC* in vitro (Figure 7G and Supplemental Figure 10A). As CUMT8^ΔpduC^ grows normally on fucose and rhamnose but not 1,2-PD (Supplemental Figure 8 and Supplemental Figure 10, C and D), and as CUMT8-WT induces *pduC*-dependent Th17 responses (15), the low levels of *pduC* transcription (Figure 7G) suggest that AIEC CUMT8 does not metabolize 1,2- PD during colonization of *IL10^–/–^* mice in the absence of DSS (15).

The enhanced virulence of CUMT8^ΔfucA^ aligns with the emerging role of fucose as a mediator of host-microbe symbiosis (4). Increased virulence of CUMT8^ΔfucA^ could not be attributed to enhanced transcription of the *eut* operon or *pduC* (Figure 7G and Supplemental Figure 10A). Loss of fucose sensing, encoded by *fusK/fusR*, is linked to increased virulence in EHEC (45), but these genes were absent in CUMT8 and present in only 1 *E*. *coli* strain (37RT-2 B2, non-AIEC) in our collection. Fucose is associated with the ability of symbiont *E*. *coli* to colonize the intestine, and deletion of *fucAO* in *E*. *coli* has been linked with enhanced ability to metabolize ribose, a sugar preferred by pathogenic *E*. *coli*, and fructose, which has been linked to intestinal inflammation and dysbiosis characterized by loss of probiotic *Lactobacillus* and *Bifidobacteria* (4, 26, 50, 51). We found that rhamnose and fructose equally supported the growth of CUMT8 and Δ*fucA*, Δ*pduC*, and Δ*eutH* derivatives (Figure 7H and Supplemental Figure 10, C–E), and they did not induce the transcription of *fucA*, *fucO*, and *pduC* (Supplemental Figure 10B). We next determined the comparative ability of fucose, rhamnose, ribose, and fructose to support the growth of patient-derived AIEC and non-AIEC. Growth on fructose and ribose was higher than fucose and rhamnose (Figure 7I), with the rate of growth for fructose approaching glucose (Supplemental Figure 10, F–H), highlighting dietary sugars, ribose, and fructose, as *fucA* independent carbon sources.

## Discussion

The primary objective of this study was to identify host and bacterial factors related to the abundance and virulence of AIEC linked to ileal CD. To address the limitations of previous studies that used fecal and in silico analyses to approximate the ileal mucosal metabolome and archival AIEC strains (21, 23, 24), we adopted a patient-based approach that combined direct analysis of the microbiome, metabolome, and cultivable bacteria of the ileal mucosa with clinical characteristics in order to guide unbiased functional analysis of AIEC and *E*. *coli* isolated from the same patients.

^1^H-NMR metabolomic analysis provided key insights into the ileal niche of AIEC, revealing differential abundance of mucosal metabolites by IBD phenotype and cosegregation of metabolites associated with inflammation, oxidative stress, and lipid peroxidation (31, 32) with CD, ileitis, and AIEC. The similar abundance of EA and acetate in the healthy and inflamed ileum of our patient cohort argues against inflammation-associated increases in EA and acetate fueling the growth of AIEC, and it mirrors findings for enteropathogens and uropathogenic *E. coli* (UPEC) (28, 41). However, cleavage of differentially abundant PC2 and PC3 and constituent PE by inflammation-associated phospholipase could liberate LPE and EA for consumption by *E*. *coli* (36, 37, 39). The mucosal metabolome of AIEC is consistent with the requirement of moderate-severe inflammation to induce outgrowth of AIEC in murine models of ileal CD, the correlation between inflammation and mucosa-associated *E*. *coli* in ileal CD (9, 18), and the association of host DNA, *Proteobacteria*, glycerophospholipids, and GSH in the feces of children with CD (52). The coabundance of creatine, adenine, and succinate — markers of impaired epithelial bioenergetics, barrier-damage, and regional hypoxia — has important implications. The creatine, phosphocreatine, and creatine kinase (Cr/CK) metabolic shuttle generates ATP to support epithelial integrity and is dysfunctional in a subset of CD patients (34, 42). Adenine and succinate are intimately related to hypoxia-inducible factors (HIFs) that have pleiotropic effects on barrier function, inflammation, and the Cr/CK shuttle (33, 34, 42). Activated neutrophils drawn to the inflamed mucosa increase oxygen demands and promote regional hypoxia, with adenine and succinate liberated by cell necrosis and with HIF expressed by the intestinal epithelium. This same process generates TTH, used by entero-pathogens as an electron acceptor to support use of EA (53), and may serve to delineate the threshold and type of inflammation that promotes outgrowth of AIEC. The potential for AIEC to modulate this hypoxic microenvironment is supported by their ability to induce NF-κB (49), which increases transcription of HIF-1α mRNA (33, 42) and induction of HIF-dependent pathways in epithelial cells by AIEC LF82 (54). However, a stepwise mechanistic understanding of the interplay between AIEC, tissue metabolism, the epithelium, and the host-immune response remains to be elucidated. While 16S rRNA analyses did not identify an AIEC-specific microbiome, changes in the functional capacity of the microbiome (30) — e.g., loss of mucolytic Bacteroides and symbiont antiinflammatory Firmicutes and enrichment in *Enterococcus* — may impact the abundance of ileal *E*. *coli* and non-AIEC. Glycerol enrichment in UC reveals a metabolic fingerprint beyond the colon that may serve as a biomarker and influence bacterial colonization — e.g., B2 *E*. *coli*.

Our discovery that ileitis and AIEC-associated metabolites support the growth of patient-derived *E*. *coli* transforms the metabolome from an inert biomarker to a nutrient-rich environment that may shape the abundance of resident *E*. *coli*, AIEC, and dysbiosis. The selective ability of AIEC to use EA as a source of nitrogen and carbon was influenced by phylogroup (greater for B1 and D), virulence gene content (greater for *pduC^+^* or *lpfA^+^*), and polymorphisms in the *eut* operon. In contrast to previous studies, there was no requirement for prior adaptation in propionate or bile salts, or inclusion of TS and TTH (21, 23, 24). We identified a network of pathways that facilitate the metabolism of EA — notably, the glyoxylate shunt, methylcitrate cycle (23), amino acid metabolism, γ-glutamyl cycle, and pleiotropic stress responses that recapitulate the arsenal employed by enteropathogens to resist nitrosative, oxidative, and superoxide stress in the inflamed gut (5, 6, 41, 44).

We discovered a multifaceted interaction between EA metabolism and amino acids, highlighted by the ability of ileitis-associated amino acids to augment the growth of AIEC on EA, which may reflect reduced glucose catabolism and enhanced secretion of acetate (46), a metabolite of the eut MCP (20); protection against oxidative and acid stress; and use of fumarate (via aspA, frdA-D, dcuC; Figure 4F) as an electron acceptor (55). This finding is also highlighted by a differential effect of Pro, stimulating growth on EA but suppressing growth on fucose and glucose (35), which may be virulence related. These findings, along with the broad suppressive effects of Phe, Met, and Leu, align with the emerging role of single amino acid utilization by different bacterial species and strains in colonization, host range, and virulence (35, 38, 46, 56, 57). The ability of AIEC to coutilize host-derived inflammation–associated amino acids and antioxidant GSH as “cofactors” to support the use of regionally available EA parallels the use of TTH/TS by enteropathogens (6, 41, 53). The differential dependency of AIEC on exogenous TS and TTH suggests that they may employ one or both of these strategies. Whether GSH induces EA ammonia lyase activity and buffers aldehyde toxicity in AIEC as described in *Salmonella* remains to be determined (58). Importantly, chemotaxis and repulsion in *E*. *coli* are also mediated by amino acids via ubiquitous chemosensors that align with ileitis-associated Asp (Tar) and regionally available Ser (Tsr) (45, 56, 59). The ability of AIEC to sense amino acids, epinephrine (*qseC*, *feaR)* (45), and potentially EA may support niche recognition, luminal-to-mucosal association, and translocation (5, 45).

Through functional analysis, we identified the ability of EA and Gln to enhance the virulence of AIEC in vitro, and we established a direct link between EA metabolism and inflammation in *IL10^–/–^* IBD-susceptible mice. The more consistent and robust effect of Gln versus EA on the invasion of epithelial cells is likely related to its superior effect on motility (24, 49). The ability of EA and Gln to enhance the survival and replication of AIECs in macrophages may reflect their value as alternate sources of carbon and nitrogen and their ability to buffer metabolic and environmental stress within the phagosome. However, the mechanism by which EA induces inflammation in *IL10^–/–^* susceptible mice is unclear, as AIEC CUMT8 does not invade epithelial cells or persist in macrophages in germ-free *IL10^–/–^* mice, and it lacks the secretion systems and pathogenicity islands regulated by EA in enteropathogens (11, 14, 19, 20). The identification of propionate generated from 1,2-PD by the *pdu* MCP as a proinflammatory effector of AIEC (25) suggests that products of the *eut* MCP, acetaldehyde, ethanol, or acetate may play a similar role (20). This is supported by the ability of ethanol and acetaldehyde to synergistically disrupt tight junctions by a mechanism involving calcium and oxidative stress (60) and the increases in these volatile organic compounds in patients with IBD (61). In the context of ileal CD, the high ability of AIEC to resist environmental stress is linked to their survival within macrophages (62), and we postulate that life in the inflamed intestine may precondition mucosa-associated and translocated AIEC to exploit defects in intracellular killing and autophagy conferred by CD risk polymorphisms (11, 12).

While our intent was to identify factors relating to the overgrowth and virulence of AIEC as an opportunistic pathogen in the inflamed ileum, our findings also provide important insights into the life of AIEC as a symbiont. The selective ability of EA to increase the growth of AIEC on fucose and the concurrent transcription of genes encoding EA and fucose/rhamnose metabolism in AIEC monoassociated *IL10^+/+^* mice support the cometabolism of fucose and EA in the healthy gut. Against this background, the enhanced virulence of CUMT8^ΔfucA^ in *IL10^–/–^* mice fits with the emerging role of fucose as a mediator of host-microbe symbiosis (4), and our findings raise the possibility that AIEC symbiosis may be facilitated by cometabolism of EA and crosstalk between the *eut* and *pdu* MCP during growth on mucosal sources of carbon and nitrogen. The consistently low transcription of *pduC* in vivo, and reciprocal changes in the transcription of *pocR* and *pduA–E* with EA in vitro, suggest that AIEC may favor the *eut* over the *pdu* MCP, the inverse of *Salmonella* (47), with the use of the *pdu* MCP determined by the luminal microenvironment (15). The ability of ileal *E*. *coli* to utilize dietary sugars, fructose, and ribose, independently of f*ucA*, has important implications for colonization and virulence when fucose availability is limited by inflammation-associated loss of mucolytic Bacteroides and mucus depletion (27, 50).

Our study has important limitations. Our patient cohort was not treatment naive or serially studied, which may have impaired the detection of AIEC-related changes in the microbiome and impacted our findings. Our ability to detect additional microbial and disease-associated signals strong enough to meet *q* < 0.2 would have been enhanced by a larger patient cohort. While the untargeted ^1^H-NMR metabolomic platform is robust and reproducible, it limits our ability to resolve glycerophospholipids, and it is not as sensitive or comprehensive as combined chromatography and mass spectrometry for many metabolites (63).

**Figure 8:**
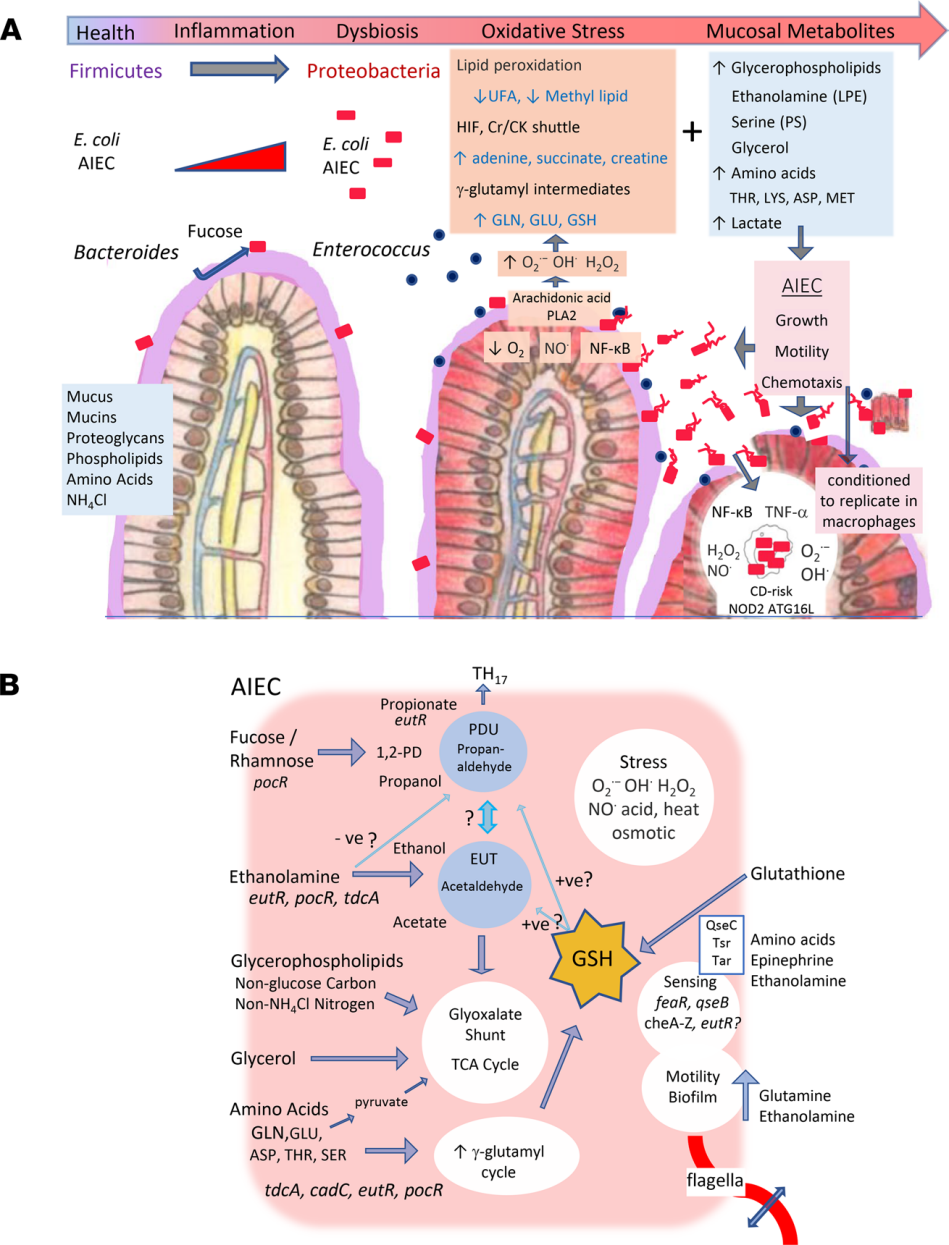
Mucosal metabolites fuel the growth and virulence of *E*. *coli* linked to Crohn’s disease. (**A**) In the healthy gut, resident AIEC can utilize a variety of mucus-derived substrates, including fucose and EA, for growth, with use of fucose/rhamnose linked to symbiosis. In the inflamed ileum, the mucosal niche of AIEC is characterized by loss of mucolytic Bacteroides and symbiont Firmicutes and culture of *Enterococcus*, oxidative stress, and an increase in metabolites associated with hypoxia and loss of barrier integrity. AIEC are better able to utilize regionally available EA than non-AIEC, and they cometabolize ileitis-associated amino acids and GSH to support metabolism of EA and resist stress in the inflamed and hypoxic mucosa. Sensing of regional metabolites may provide spatial awareness and induce chemotaxis to facilitate growth and mucosal association or translocation. In an IBD susceptible host, EA may enhance the ability of AIEC to induce inflammation. The ability of AIEC to resist environmental and metabolic stress in the inflamed intestine may precondition translocating AIEC for survival in macrophages. (**B**) Metabolic plasticity of AIEC is enabled by the *eut* and *pdu* MCP, glyoxylate shunt, methylcitrate pathway, pleiotropic stress responses, amino acid degradation, and γ-glutamyl-cycle. Sensing of amino acids, epinephrine, and ethanolamine modulates motility, chemotaxis, and biofilm formation.

Collectively, we identified the mucosal metabolome as a dominant factor related to the culture of AIEC, and we discovered that AIEC are pathoadapted to utilize mucosal metabolites linked to health and inflammation and regionally available EA for growth, energy, stress resistance, and virulence (Figure 8A). This metabolic plasticity, facilitated by the *eut* and *pdu* MCPs, the glyoxylate shunt, methylcitrate cycle, amino acid metabolism, γ-glutamyl-cycle, and pleotropic stress responses (Figure 8B) may underpin the ability of AIEC to colonize the healthy and inflamed intestine and to transition from symbiont to pathogen in a susceptible host. From a clinical perspective, we highlight potential biomarkers of AIEC and identify metabolic pathways as novel targets for therapeutic intervention against AIEC and *E*. *coli*-associated dysbiosis.

## Methods

### Patients.

A cohort of 97 patients, including 43 CD, 25 UC, and 29 non-IBD, was studied. The presence of CD risk–associated polymorphisms in NOD2 (R702W, G908R, 1007FS) and ATG16L (rs2241880; leading to a T300A conversion: GG, AG, AA) was determined at the Wellcome Trust Clinical Research Facility in Edinburgh as described previously for ATG16L (64) and Supplemental Methods for NOD2.

### Bacterial culture and characterization.

Bacteria were isolated from ileal biopsies, and *E*. *coli* was characterized for genotype (RAPD-PCR), phylogroup, virulence genes including diarrheagenic *E*. *coli* virulence genes, and gentamicin resistance as previously described (9, 19).

### Cell lines and culture conditions.

The human colonic epithelial cell line Caco-2 (ATCC HTB-37), murine macrophage cell line J774A.1 (ATCC TIB-67), and HEK-Blue KD-TLR5 cell line (InvivoGen) were used in the study, with culture conditions described previously (9, 49).

### Evaluation of the AIEC pathotype.

The ability of *E*. *coli* strains to invade Caco-2 epithelial cells and survive in J774 macrophages was determined as previously described (9).

### NF-κB activation assay.

HEK-Blue KD-TLR5 cells were used to detect the induction of NF-κB by *E*. *coli* infection as described previously (49).

### Motility assay.

*E*. *coli* was grown overnight at 37°C in LB broth. Soft agar plates (1% tryptone, 0.5% NaCl, 0.25% agar ± additional substrates as specified in the results) were prepared the day before the assay, and motility was assessed as previously described (49).

### 16S rRNA-Seq and analysis.

DNA was extracted from the ileal biopsies and amplified with primers targeting the 16S rRNA gene V4 variable region F515/R806, and it was sequenced with Roche 454 FLX titanium instruments as previously described (18). The complete source code for this analysis and raw sequencing data is available at https://gitlab.com/morganlab/Simpson2021 and http://www.ncbi.nlm.nih.gov/bioproject/781964 Detailed methodology is in Supplemental Methods.

### ^1^H-NMR spectroscopic analysis of intact ileal biopsies.

Intact snap-frozen ileal biopsies were bathed in ice-cold saline D_2_O solution. A portion of the tissue (~15 mg) was inserted into a zirconium oxide (ZrO_2_) 4 mm outer diameter rotor using an insert to make a spherical sample volume of 25 μL. The tissue NMR spectra were converted into 2000 data points over the range of δ 0.0–10.0 using an in-house MATLAB routine excluding the water residue signal. Chemical shift intensities were normalized to the sum of all intensities within the specified range prior to chemometric analysis for tissue samples (detailed in Supplemental Methods). The complete source code for this analysis and raw data are available at https://gitlab.com/morganlab/Simpson2021

### Growth of E. coli in chemically defined media.

For growth experiments, we evaluated 54 *E*. *coli* strains: 50 from human ileal mucosa, including 11 that we have described previously (9) and prototypical AIEC LF82 (10), murine AIEC NC101 (14), and CUMT8 (18), as well as AIEC 2A from CD feces (15) (Supplemental Table 4). Two types of culture media were used for this study, Luria-Bertani (LB) and chemically defined M9 minimal media. The composition of M9 minimal medium was described previously (49). Different combinations of carbon and nitrogen sources were used and specifically described in the figure legends, including 4 carbon sources (20 mM glucose, 20 mM fucose, 20 mM glycerol, and 20 mM EA) and 3 nitrogen sources (19 mM NH_4_Cl, 20 mM EA, and 2.5 mM Gln). *E*. *coli* growth curves were generated with the Bioscreen C System (Growth Curves USA) under microaerophilic conditions as described previously (49). The AUC was calculated using GraphPad Prism 7.03 software.

### EA operon alignment and gene content of E. coli.

Sequences of the *eut* operon for 55 *E*. *coli* strains in our collection were provided by Enterome. Alignments of the EA operon were performed using the Geneious Prime software. The sequences of alignments are available in GenBank (BankIt2520789 and BankIt2522416). The presence or absence of *norV*, *soxR*, *gadE*, *rpoH*, *tdcA*, *cadC*, *ykgA*, *FeaR*, *hcaR*, *fusK*, and *fusR* was determined by Geneious Prime software using reference gene sequences from *E*. *coli* K12 MG1655 (CP027060.1) for all genes except *fusK/R*, which were from *E*. *coli* O157:H7 (CP039837.1).

### Metatranscriptomic analyses of AIEC CU541-1.

M9 minimal media with specified carbon and nitrogen sources was inoculated with overnight cultures (at 1:50 dilution) of AIEC CU541-1 and incubated under microaerophilic conditions (5% CO_2_, 5% O_2_) at 37°C without shaking until the mid-log phase. RNA isolation, library construction, and sequencing are described in the Supplemental Methods. The raw sequencing data are available at https://submit.ncbi.nlm.nih.gov/subs/bioproject/SUB10604108/overview

### Construction of CUMT8 derivative strains.

An isogenic mutant of CUMT8 lacking the complete *eutH* gene was constructed using the λ red recombinase system using deletion primers as described previously (19). Primer sequences and procedures are provided in the Supplemental Methods.

### Animal models.

Eight- to 12-week-old germ-free (GF) 129SvEv *Il10*^−/−^ and WT mice were housed at the UNC National Gnotobiotic Rodent Resource Center. An overnight-cultured *E*. *coli* strain (5 × 10^8^ to 8 × 10^8^ bacteria/mL) was inoculated into GF mice by oral and rectal swabbing in gnotobiotic isolators under a strict 12-hour light/dark cycle and fed an autoclaved, polysaccharide-rich, standard rodent chow diet (14). Analytical procedures are described in the Supplemental Methods.

### Targeted transcriptional analysis of AIEC.

The expression of bacterial virulence and metabolic genes was determined by reverse transcription PCR (RT-PCR). Total RNA was extracted from the cecal content of monoassociated mice using the RNeasy Power Microbiome Kit (Qiagen) or from *E*. *coli* cultures grown in chemically defined media as previously described (49). RT-PCR was performed as described previously (49), with primers and conditions given in the Supplemental Methods.

### Statistics.

Statistical procedures for the integrated analysis of patient phenotype, genotype, microbiome, and metabolome are described in Supplemental Methods. For other experiments, data are presented as the mean ± SEM. Significance was determined using 2-tailed, unpaired Student’s *t* tests for 2 groups; 2-way ANOVA corrected for multiple comparisons with Tukey’s posttest. *P* values less than 0.05 were considered significant. Prism 9 software (GraphPad) was used for statistical analysis.

### Study approval.

All patients provided signed informed consent to submit ileal mucosal biopsies to the Tissue Bank (protocol no. 0603-859). Ileal biopsy samples were collected endoscopically, and inflammation was determined by endoscopic scoring and histopathology as previously described (9). This study was approved by the Cornell University Committee on Human Subjects (protocol no. 05-05008).

## Author contributions

Conceptualization was contributed by KWS, RBS, and EJS. Clinical cohort (patient phenotypes and biological samples) was contributed by EJS, RL, and RY. Bacterial culture and characterization were contributed by BD, SZ, and EID. 16S sequencing was contributed by SED. ^1^H-NMR metabolomics was contributed by FPM. Transcriptomics was contributed by LAM, SS, and SZ. Genomics of *E*. *coli* was contributed by MP and BD. CD risk genotyping was contributed by JS. Murine models were contributed by AO, BL, JH, and RBS. Integrated analysis was contributed by SZ, XM, SS, CH, BD, and KWS. Resources were contributed by KWS, RBS, FPM, CH, and EJS. Data curation was contributed by SZ, XM, SS, LAM, and MP. Writing of the original draft was contributed by SZ, KWS, XM, and BD. Visualization was contributed by SZ, XM, SS, BD, AO, and KWS. Funding acquisition was contributed by KWS, RBS, RL, and EJS.

## Supplementary Material

Supplemental data

Supplemental tables 1-21

## Version 1 04/12/2022

In-Press Preview

## Version 2 05/23/2022

Electronic publication
