# Supplementary material for: Mucosal metabolites fuel the growth and virulence of E. coli linked to Crohn’s disease
Source: JCI Insight. 2022 May 23;7(10):e157013. doi: 10.1172/jci.insight.157013 (PMC9220930; doi:10.1172/jci.insight.157013)
Supplement: Supplemental data [file jciinsight-7-157013-s252.pdf]

Supplemental methods

NOD2 Genotyping. Genotyping of NOD2 variants (R702W, G908R and 1007FS) was performed by high resolution melt analysis on a Rotor-Gene 6000 (Corbett Life Science). The primer sequence and expected product size were:

R702W (F: 5'-CTGGCTGAGTGCCAGACATC-3' and R: 5'-GGATGGAGTGGAAGTGCTTG-3', 100 bp)

G908R (F: 5'-AGGCCACTCTGGGATTGAG-3' and R: 5'-GTGATCACCCAAGGCTTCAG-3', 195 bp)

1007FS (F: 5'-GGCAGAAGCCCTCCTGCAGGGC-3' and R: 5'-CCTCAAAATTCTGCCATTCC-3', 150 bp).

PCR was carried out in a 20 µl reaction volume containing: 10 µl Invitrogen Express SYBR Green qPCR supermixes, 900 nM forward and reverse primers and DNA template up to 120 ng. All samples were amplified in duplicates and each run included genomic DNA from sequence verified wild-type, heterozygous and homozygous genotypes for each of the variants. Data were analyzed using the manufacturer's software. All raw data were normalized by selecting linear regions before and after the melting transition, and the resulting melt curve profiles were compared to melting profiles from known positive control samples included in the same run.

16S rRNA sequencing and analysis. PCR products from different samples were mixed in equal concentrations and purified using Agencourt Ampure beads (Agencourt Bioscience Corporation). Samples were sequenced using Roche 454 FLX titanium instruments and reagents following manufacturer's guidelines. Mothur (1) was used to generate fastq files from .fasta and .qual files, and Data2 (2) was used to analyze 16S rRNA sequencing data. Taxonomy was assigned using the Silva database (v1.32) (3). The packages phyloseq (4), vegan (5), and rstatix were used for statistical analysis. For alpha diversity analysis, data was rarefied to 2000 reads per sample. Anova and Tukey's HSD test were used to test for differences in alpha diversity between disease groups. Kruskal-Wallis tests with Benjamini-Hochberg false discovery correction were used to test for associations between taxa and metadata of interest. Data was normalized to

relative abundance and glommed to genus level prior to Kruskal-Wallis testing and beta diversity analysis. The threshold for false discovery was $q < 0.25$ for all results. Kruskal-Wallis testing was followed by Dunnet's posthoc testing when metadata included multiple categories. The Bray-Curtis distance was used for all beta diversity analysis. The complete source code for this analysis and raw sequencing data is available at https://gitlab.com/morganlab/Simpson2021. The raw sequencing data can also be found at http://www.ncbi.nlm.nih.gov/bioproject/781964.

^{1}H NMR spectroscopic analysis intact ileal biopsies. Intact snap-frozen ileal biopsies were bathed in ice cold saline D_2O solution. A portion of the tissue (~15 mg) was inserted into a zirconium oxide (ZrO_2) 4 mm outer diameter rotor, using an insert to make a spherical sample volume of 25 µl. In order to reduce NMR spectral peak broadening caused by any residual dipolar couplings, chemical shift anisotropy and microscopic inhomogeneities, 600 MHz ^{1}H NMR spectra of intact tissues were acquired with a high resolution magic-angle-spinning probe at a spin rate of 5000 Hz. ^{1}H NMR spectra were acquired for each sample using a Bruker Avance II 600 NMR spectrometer (Bruker Biospin, Rheinstetten). Tissue samples were regulated at 283K using cold N_2 gas during the acquisition of the spectra to minimize any time-dependent biochemical degradation, as previously described (6). Standard ^{1}H NMR one-dimensional pulse sequence with water suppression, Carr-Purcell-Meiboom-Gill (CPMG) spin-echo sequence with water suppression, and diffusion-edited sequence were acquired for sample, as described previously (6). For each sample, 256 and 16 scans were collected into 98'000 data points using a spectral width of 18028 Hz and 18315 Hz, respectively. The assignment of the ^{1}H-NMR resonances to specific metabolites was achieved by matching our in-house NMR database of pure compounds and using literature data.

The tissue NMR spectra were converted into 22K data points over the range of δ 0.0-10.0 using an in-house MATLAB routine excluding the water residue signal. Chemical shift intensities were normalized to the sum of all intensities within the specified range prior to chemometric analysis for tissue samples.

Chemometric analysis was performed using the software package SIMCA-P+ (version 12, Umetrics AB, Umeå, Sweden) and in-house MATLAB (The MathWorks Inc., Natick, MA, USA) routines.

One glucose peak was omitted from downstream analysis due to suspected lipid contamination. Samples with negative integral values for a metabolite were omitted from analysis of that metabolite. Kruskal-Wallis tests with Benjamini-Hochberg false discovery correction and permutation-based ANOVA were used to test for associations between metabolites and metadata of interest. Each metabolite – metadata association with uncorrected $p < 0.05$ was included in visualization, and its effect size (η^2 / eta squared) was calculated. The packages FactoMineR and Factoextra were used for principal components analysis to visualize relationships between metabolites and phenotypes. The complete source code for this analysis and raw data is available at https://gitlab.com/morganlab/Simpson2021.

Meta-transcriptional analyses of AIEC CU541-1. M9 minimal media with specified carbon and nitrogen sources was inoculated with overnight cultures (at 1:50 dilution) of AIEC 541-1 and incubated under microaerophilic conditions (5% CO_2, 5% O_2) at 37ºC without shaking until the mid-log phase. Total RNA was isolated with RNAProtect Reagent and RNeasy Kit (Qiagen) per manufacturer's protocols. The trace amount of gDNA contamination in the total RNA samples was removed with TURBO DNA-free Kit (Ambion), as described in the manufacturer's protocol. The RNA-seq libraries were constructed with the Complete Library Kit for bacteria (Epicentre) per manufacturer's instructions. The libraries were sequenced using Illumina Hiseq 2000 platform at Cornell Biotech Genome facility with paired-end reads (50 bp) Reads cleaned and trimmed based on a quality score of 25 and minimum length of 25 bp with fastq-mcf (https://code.google.com/p/ea-utils/wiki/FastqMcf). Paired-end reads from fragments shorter than twice the read length were merged with Flash (7). RNAmmer (8) and tRNAscan (9) were used to find ribosomal RNAs and tRNAs, respectively. Predicted proteins were queried against Swiss-Prot database (10) with BLASTx to determine putative functions. Interproscan (11) was used to assign GO terms and domains. COGs were assigned by BLAST matches to the cog database at

ftp://ftp.ncbi.nih.gov/pub/mmdb/cdd/big_endian/. Cleaned reads were mapped to the new genome assemblies with Tophat2 (12). Read counts to gene models were calculated using the htseq package (13) and FPKM was calculated using cuffdiff (14). Differential expression was calculated using edgeR and cuffdiff. Heatmaps were generated using the R heatmap.2 package and custom scripts. The raw sequencing data is available at https://submit.ncbi.nlm.nih.gov/subs/bioproject/SUB10604108/overview.

Construction of CUMT8 derivative strains. An isogenic mutant of CUMT8 lacking the complete eutH gene was constructed using the λ red recombinase system using deletion primers as described previously (15). Primers contain sequences homologous to the 5′ and 3′ ends of eutH: eutH-delF (5′-GCATCCGCCAATAAATAACAACTAACAGGGAGTAAAGGCGGTGTAGGCTGGAGCTGCTTC-3′); and eutH-delR (5′-CGATATCGATACCGACGCTCAATAGCTGGCGAGTGTTCACCATATGAATATCCTCCTTAG -3′) were used to amplify the resistance cassette from the template plasmid pKD4 encoding kanamycin resistance. PCR products were electroporated into CUMT8 containing λ red recombinase plasmid pKD46 after which kanamycin-resistant colonies were selected. The deletion of eutH was confirmed by PCR. fucA and pduC mutants were created similarly as described previously (15). To complement the eutH mutation, the complete eutH coding region was amplified by PCR from CUMT8, restriction digested and cloned into the kpnI and hindIII sites of plasmid pBBR1MCS. This plasmid construct was electroporated into CUMT8:ΔeutH and selected for resistance to chloramphenicol. Constructs were confirmed by PCR and sequencing using primers flanking the insertion sites.

Animal Models. Eight to twelve week-age germ-free (GF) 129SvEv background IL-10-deficient (*Il10*$^{-/-}$) and wild-type (WT) mice were obtained from UNC National Gnotobiotic Rodent Resource Center. Overnight-cultured CUMT8 wild-type, CUMT8$^{\Delta eutH}$, CUMT8$^{\Delta pduC}$ or CUMT8$^{\Delta fucA}$ strain was inoculated to

GF mice by oral and rectal swabbing in gnotobiotic isolators under a strict 12 h light cycle and fed an autoclaved, polysaccharide-rich, standard rodent chow diet. Colonization was confirmed with both RAPD-PCR and *eutH, pduC* and *fucA* specific PCRs using DNA extract of the fecal content using primers:

eutH-F (5′-CGGTGGCGCTCGGCATTATC-3′), *eutH*-R (5′-CAGCGAGTCAGAAGCAGCACCATT-3′)

fucA-F (5′-GGCAACGGTAAACATGAGGAAGGA-3′), *fucA*-R (5′-TAATCGCCAGGGTCGTCAGGTAAA-3′)

pduC-F (5′-GCGCCCGTCGAACACCATCAC-3′) and *pduC*-R (5′-TTGCCTTCCGGCGTAACCCATCTGT-3′)

Twelve weeks after colonization, colon tissues were harvested and analyzed by histological score, cytokine qPCR, or tissue fragment culture following previously published protocols (16–18). All procedures were performed at UNC and approved by the UNC Institutional Animal Care and Use Committee.

Quantitative PCR for mucosal cytokines. Between 15−20 mg of tissues stored in RNAlater (Qiagen) were used and the tissue RNA extractions were performed with RNeasy Plus Mini kit (Qiagen) according to the manufacturer's protocols. cDNA was created with the SensiFAST cDNA synthesis kit (Bioline) by PCR (25°C, 10 min; 42°C, 15 min; 85°C, 5 min). Quantitative PCR was performed with QuantStudio3 (Thermo Fisher Scientific, PA, USA) using SYBR No-ROX reagents (Bioline) with the following PCR setting: 95°C, 2 min; 95°C, 5 sec; 40 cycles of (60°C, 10 sec; 72°C, 20 sec); melting curve analysis: 95°C, 15 sec; 60°C, 15 sec; 95°C, 15 sec. The data were created by comparative Ct method ($2^{-\Delta\Delta Ct}$). The following validated PCR primers were used:

IFNg (5'-CTTCCTCATGGCTGTTTCTGG-3') and (5'-ACGCTTATGTTGTTGCTGATGG-3')

Il12b (5'-CGCAAGAAAGAAAAGATGAAGGAG-3') and (5'-TTGCATTGGACTTCGGTAGATG-3')

Il23a (5'-GCCCCGTATCCAGTGTGA-3') and (5'-GCTGCCACTGCTGACTAGAA-3')

Il17a (5'-CTCAGACTACCTCAACCGTTC-3') and (5'-TGAGCTTCCCAGATCACAGAG-3')

Actb (5'-AGCCATGTACGTAGCCATCCAG-3') and (5'-TGGCGTGAGGGAGAGCATAG-3').

Each cDNA sample was analyzed in duplicate for quantitative assessment of RNA amplification. Melting curve analysis confirmed the presence of single products with expected melting temperatures. Expression of housekeeping gene Actb was more consistent than that of GAPDH and 18S between tissues in the model used in this study.

Targeted transcriptional analysis of AIEC. RT-PCR primers for virulence, metabolic and stress genes are listed in Table S1. *E. coli mdh* was used as the reference gene. Each RT-PCR reaction contained 1 µl of cDNA, 0.7 µl of each forward and reverse primers (10 µM), 5 µL of 2X SYBR Green Master Mix (Qiagen), 1 µl of QN ROX Reference Dye and 2.3 µl of nuclease-free water to make the total volume of 10 µl. The reaction was run with ABI7000 (Applied Biosystems). Differential transcription (ΔCt) was determined by subtracting the average Ct of the target gene from the average Ct of housekeeping gene (*mdh*).

Table S1 RT-PCR primers used in this study

Gene	Forward (5' ---> 3')	Reverse (3' ---> 5')	Function
pduC	TGAAGAAGTCGAAGCGGCAACCTA	TTTGCATGTTGAGCATGTCCTGGG	Fucose metabolism
fucA	CGACCTTTGGAACACGCGAACTTT	TCACCTCACAAGCGATAAGCCCAT	Fucose metabolism
fucO	TGAACGAAACGGCATGGTTTGGTC	ACGATCAGCGCCTTCTGATAACCA	Fucose metabolism
eutR	CCGGAAAATCTCCATCAGCCTG	CTGATGACTGATGCTTTCCGCC	Ethanolamine metabolism
eutB	TTCGCTGATGCTCCAGTTT	CCGGTGATTGCCTATGAAGAT	Ethanolamine metabolism
eutC	TGCTGGAAGTACGCTCCGAAAT	CCGGATTAGCAACACACTGTGC	Ethanolamine metabolism
eutE	AAAATATCGACGAGCGCGGAAA	CCACAAACAGCAAACGCGTTTC	Ethanolamine metabolism
eutH	CGCTCGGCATTATCGAACCTTC	GCACACCGGAGTACATAGCAAC	Ethanolamine metabolism
gltB	GCGAACCTAGCCACAAGGTAGT	GTTTTTGTAACAGCAAGCCGCA	Glutamate synthase
glnA	CGGCTGGAAAGGCATTAACGAG	CCAGGTTCAAGGATGTCGCAAC	Glutamine synthatase
gshA	AAAGCGTTTGCAGAAGCCTACC	GGTTTCCATTTCCTGCTGACGG	Glutathione synthatase

gshB	AAAGTGACTGGAAAATCGCCCG	AGGTTGGGCTGGTGACGTTAAT	Glutathione synthatase
rpoS	CCACTGTTAACGGCCGAAGAAGAA	CGCCAGACCACGATTGCCATAA	Stress response
uidA	TCAACAGACGCGTGGTTACAGTCT	TCCATCGCAGCGTAATGGTCTACA	Stress response
fliC	TCTCTGAGCTCCGCCATTGAA	TCGCGTTACGGGAAGCCTGAG	Flagellar synthesis
mdh	TGGCCAGGCGCTTGCACTAC	GCGCCGGAGTCGCATCTTCA	House keeping gene

References:

1. Schloss PD et al. Introducing mothur: Open-source, platform-independent, community-supported software for describing and comparing microbial communities. *Appl. Environ. Microbiol.* 2009;75(23). doi:10.1128/AEM.01541-09

2. Callahan BJ, Sankaran K, Fukuyama JA, McMurdie PJ, Holmes SP. Bioconductor Workflow for Microbiome Data Analysis: from raw reads to community analyses. *F1000Research* 2016;5. doi:10.12688/f1000research.8986.2

3. Pruesse E et al. SILVA: A comprehensive online resource for quality checked and aligned ribosomal RNA sequence data compatible with ARB. *Nucleic Acids Res.* 2007;35(21). doi:10.1093/nar/gkm864

4. McMurdie PJ, Holmes S. Phyloseq: An R Package for Reproducible Interactive Analysis and Graphics of Microbiome Census Data. *PLoS One* 2013;8(4). doi:10.1371/journal.pone.0061217

5. Oksanen J et al. Vegan: Community Ecology Package. R package version 2.5-7.. *Community Ecol. Packag.* 2020;10.

6. Martin FPJ et al. Panorganismal gut microbiome-host metabolic crosstalk. *J. Proteome Res.* 2009;8(4). doi:10.1021/pr801068x

7. Magoč T, Salzberg SL. FLASH: Fast length adjustment of short reads to improve genome assemblies. *Bioinformatics* 2011;27(21). doi:10.1093/bioinformatics/btr507

8. Lagesen K et al. RNAmmer: Consistent and rapid annotation of ribosomal RNA genes. *Nucleic Acids Res.* 2007;35(9). doi:10.1093/nar/gkm160

9. Lowe TM, Eddy SR. TRNAscan-SE: A program for improved detection of transfer RNA genes in genomic sequence. *Nucleic Acids Res.* 1996;25(5). doi:10.1093/nar/25.5.0955

10. Bateman A et al. UniProt: A hub for protein information. *Nucleic Acids Res.* 2015;43(D1). doi:10.1093/nar/gku989

11. Zdobnov EM, Apweiler R. InterProScan - An integration platform for the signature-recognition methods in InterPro. *Bioinformatics* 2001;17(9). doi:10.1093/bioinformatics/17.9.847

12. Kim D et al. TopHat2: Accurate alignment of transcriptomes in the presence of insertions, deletions and gene fusions. *Genome Biol.* 2013;14(4). doi:10.1186/gb-2013-14-4-r36

13. Anders S, Pyl PT, Huber W. HTSeq-A Python framework to work with high-throughput sequencing data. *Bioinformatics* 2015;31(2). doi:10.1093/bioinformatics/btu638

14. Trapnell C et al. Transcript assembly and quantification by RNA-Seq reveals unannotated transcripts and isoform switching during cell differentiation. *Nat. Biotechnol.* 2010;28(5). doi:10.1038/nbt.1621

15. Dogan B et al. Inflammation-associated adherent-invasive Escherichia coli are enriched in pathways for use of propanediol and iron and M-cell translocation. [Internet]. *Inflamm. Bowel Dis.* 2014;20(11):1919–32.

16. Schmitz JM et al. Murine Adherent and Invasive E. coli Induces Chronic Inflammation and Immune Responses in the Small and Large Intestines of Monoassociated IL-10-/- Mice Independent of Long Polar Fimbriae Adhesin A. *Inflamm. Bowel Dis.* 2019;25(5). doi:10.1093/ibd/izy386

17. Kim SC et al. Variable phenotypes of enterocolitis in interleukin 10-deficient mice monoassociated with two different commensal bacteria. *Gastroenterology* 2005;128(4):891–906.

18. Mishima Y et al. Microbiota maintain colonic homeostasis by activating TLR2/MyD88/PI3K signaling in IL-10-producing regulatory B cells.. *J. Clin. Invest.* 2019;130:3702–3716.
